# Enriched circulating and tumor-resident TGF-β^+^ regulatory B cells in patients with melanoma promote FOXP3^+^ Tregs

**DOI:** 10.1080/2162402X.2022.2104426

**Published:** 2022-07-28

**Authors:** Robert J Harris, Zena Willsmore, Roman Laddach, Silvia Crescioli, Jitesh Chauhan, Anthony Cheung, Anna Black, Jenny L. C. Geh, Alastair D MacKenzie Ross, Ciaran Healy, Sophia Tsoka, James Spicer, Katie E Lacy, Sophia N Karagiannis

**Affiliations:** a St. John’s Institute of Dermatology, School of Basic & Medical Biosciences, King’s College London, Guy’s Hospital, London, UK; b King’s Health Partners Cancer Research UK Cancer Centre, King’s College London, London, UK; c Department of Informatics, Faculty of Natural, Mathematical and Engineering Sciences, King’s College London, London, UK; d Breast Cancer Now Research Unit, School of Cancer & Pharmaceutical Sciences, King’s College London, Guy’s Hospital, London, UK; e Department of Plastic Surgery at Guy’s, King’s, and St. Thomas’ NHS Foundation Trust, London, UK; f School of Cancer & Pharmaceutical Sciences, King’s College London, Guy’s Hospital, London, UK

**Keywords:** Melanoma, Tumor-infiltrating lymphocytes, B cell, regulatory B cell, inflammatory B cell, regulatory T cell, immune checkpoint, tumor microenvironment, TGF-β, TNF-α

## Abstract

B cells are emerging as key players of anti-tumor adaptive immune responses. We investigated regulatory and pro-inflammatory cytokine-expressing B cells in patients with melanoma by flow cytometric intracellular cytokine, CyTOF, transcriptomic, immunofluorescence, single-cell RNA-seq, and B:T cell co-culture analyses. We found enhanced circulating regulatory (TGF-β^+^ and PD-L1^+^) and reduced pro-inflammatory TNF-α^+^ B cell populations in patients compared with healthy volunteers (HVs), including lower IFN-γ^+^:IL-4^+^ and higher TGF-β^+^:TNF-α^+^ B cell ratios in patients. TGF-β-expressing B cells in the melanoma tumor microenvironment assembled in clusters and interacted with T cells via lymphoid recruitment (SELL, CXCL13, CCL4, CD74) signals and with Tregs via CD47:SIRP-γ, and FOXP3-promoting Galectin-9:CD44. While reduced in tumors compared to blood, TNF-α-expressing B cells engaged in crosstalk with Tregs via TNF-α signaling and the ICOS/ICOSL axis. Patient-derived B cells promoted FOXP3^+^ Treg differentiation in a TGF-β-dependent manner, while sustaining expression of IFN-γ and TNF-α by autologous T-helper cells and promoting T-helper cell proliferation *ex vivo*, an effect further enhanced with anti-PD-1 checkpoint blockade. Our findings reveal cytokine-expressing B cell compartments skewed toward regulatory phenotypes in patient circulation and melanoma lesions, intratumor spatial localization, and bidirectional crosstalk between B and T cell subsets with immunosuppressive attributes.

## Introduction

Melanoma is considered a highly immunogenic malignancy, evidenced by systemic and local activation of immune responses, significant infiltration of immune cells, and high neoantigenic load.^1^ Despite this, studies into the immune response to melanoma have identified several features including regulatory T cells (Tregs) likely to contribute to immune escape. B cells have been historically considered as positive regulators of immune responses. Through differentiation into antibody-secreting plasmablasts and plasma cells, and by acting as professional antigen presenting cells, B cells can prime and activate CD4^+^ and CD8^+^ T cells, and may promote the clearance of pathogens or malignant cells.^2–4^ In contrast, there is also emerging evidence for the role of B cells in the regulation of immune responses, with autoantibody expression found to be associated with disease burden.^5^ IL-10-expressing regulatory B cells (Bregs) have been reported in patients with multiple sclerosis,^6^ systemic lupus erythematosus,^7^ and rheumatoid arthritis,^8^ and data from mouse models also indicate that IL-10^+^ Bregs could contribute to tumor progression.^9^

Besides IL-10-expressing B cells, additional subsets of regulatory B cells have been identified in humans. Naïve PD-L1-expressing B cells are reported to be upregulated in patients with advanced melanoma, which functions by curtailing T cell responses *ex vivo*.^10^ A novel IgG4^+^ CD49b^+^ CD73^+^ B cell subset has been reported in the peripheral blood and tumors of melanoma patients, which expressed pro-angiogenic and inflammatory mediators including VEGF, CYR61, ADM, FGF2, PDGFA, and MDK, and promoted endothelial cell tube formation *in vitro*.^11^ Alongside the presence of B cells expressing regulatory cytokines and inhibitory ligands, B cells expressing pro-inflammatory mediators, including TNF-α, remain poorly explored. One study reported that B cells from melanoma patients' peripheral blood express TNF-α and/or IL-6, and TNF-α transcripts from single B cells extracted from melanoma metastases were associated with reduced responsiveness to checkpoint inhibitor immunotherapy.^12^

Although features reflecting a perturbed regulatory B cell landscape have been reported in cancers^13^ including breast,^14,15^ gastric,^16,17^ and head and neck squamous cell carcinoma,^18^ the systemic and intratumoral regulatory B cell landscape in cancer patients has not yet been thoroughly explored, most likely represents multiple immunomodulatory properties, B cell lineages, and phenotypes, and may differ among tumor types. Furthermore, more broad investigations into the phenotype and function of TGF-β or IL-10-producing regulatory and IFN-γ- or TNF-α-expressing inflammatory B cells in human melanoma are still lacking.

Here, by intracellular cytokine assays, multicolor flow, and cytometry by time of flight (CyTOF), immunohistochemistry/immunofluorescence (IHC/IF), *ex vivo* co-culture studies, coupled with single-cell RNA-seq and bulk gene expression data analyses, we investigated cytokine-expressing (TGF-β, IL-4, IL-10, IFN-γ, and TNF-α) B cells in the circulation of patients with melanoma and healthy volunteers, and in human melanoma lesions. We studied evidence of interactions between cytokine expressing B cells with conventional (Tcon) and regulatory (Treg) T cell populations. In the co-culture assays, we evaluated the potential of melanoma patient B cells to mediate modulation of autologous CD4^+^ T-helper cell phenotype and functions, including the modulation of T cell proliferation, TNF-α, IFN-γ, and FOXP3 expression and induction of FOXP3^+^ Tregs.

## Materials and methods

### Human tissue samples

Human venous blood samples were collected from healthy volunteers (HV); blood and cancer lesion specimens were sourced from patients with melanoma. Peripheral blood mononuclear cells (PBMCs) were isolated from venous whole blood samples using Ficoll-Paque PLUS density gradient centrifugation, within 24 hours of phlebotomy. Single-cell suspensions were prepared from melanoma specimens as previously described.^19^ All samples were collected in accordance with the Human Tissue Act 2006 and with informed written consent in accordance with the Helsinki Declaration. This study was approved by the Guy’s Research Ethics Committee, Guy’s and St Thomas NHS Trust. The studies were conducted at King’s College London, Guy’s and St Thomas’ NHS Foundation Trust: 08/H0804/139 approved by the London Bridge NRES committee; 16/LO/0366, approved by the London-Central NRES Committee. Healthy volunteer and melanoma patient clinical information is described in **Supplementary Table 1 and Supplementary Table 2**.

### CyTOF phenotyping

For CyTOF (Cytometry by Time of Flight) phenotypic analysis, 34 metal-tagged antibodies were used to stain frozen PBMCs from 26 melanoma patients and 12 age-matched HV. Antibodies included were specific to cell surface and intracellular markers (stained separately) with a focus on B cell phenotyping. Cells were stained with a mixture of commercially available antibodies from Fluidigm®, and in-house conjugated antibodies. Files (.fcs) were processed and normalized using Fluidigm® (CyTOF normalization software 2). The CD19 population of interest was manually gated in FlowJo (illustrated in Supplementary Figure 1) and subsequently .fcs files were uploaded in R. A selection of 19 B cell directed phenotypic markers were subsequently used to perform in-depth B cell phenotyping to allow downstream identification of TGF-β and PD-L1 expressing regulatory B cells. We used a modified R script based on the CATALYST, diffCYT, FlowSOM, edgeR, and flowCORE packages, which can be found using the bioconductor terminal https://www.bioconductor.org/packages/release/bioc/vignettes/CATALYST/inst/doc /differential.html. Unsupervised clustering of B cell populations was performed using FlowSOM package to generate aggregates of B cells with phenotypic similarities. High dimensionality reduction was performed for data visualization in a 2D plot using a UMAP (Uniform Manifold Approximation and Projection) algorithm.^20^ Further details concerning cell staining, quantification, and data analysis can be found in **Supplementary Materials and Methods**.

### Intracellular cytokine assay

A flow cytometry-based intracellular cytokine assay was used to investigate the cytokine expression profiles among key B cell subpopulations in HV and melanoma patient peripheral blood, and tumor specimens. First, PBMCs were isolated from peripheral blood, and single cells isolated from melanoma lesions. Cell suspensions (4x10^6^ cells/ml) were cultured in sterile DMEM (10% Fetal Bovine Serum (FBS) and 50 U/ml Pen-Strep; Thermo Fisher Scientific) containing up to four conditions, in order to capture Breg and inflammatory B cell populations responding via innate^2^ and adaptive^7^ pathways: 0.1 µg/ml CpG ODN 2006 (Miltenyi Biotec), 10 µg/ml CpG ODN 2006, 1 µg/ml CD40L (BioLegend), or both 1 µg/ml CD40L and 10 µg/ml CpG ODN 2006. For each condition, 500 µl per well was added to flat-bottom 24-well plates and plates were incubated at 37°C with 5% CO_2_ for 72 hours. TGF-β-expressing B cells were analyzed in human blood samples without the requirement for *ex vivo* cellular activation, since TGF-β expression was detected in freshly isolated cells and our analyses showed TGF-β expression was reduced with additional *ex vivo* activation (Supplementary Figure 2). For the detection of IL-10, IL-4, IFN-γ and TNF-α, Cell Activation Cocktail with Brefeldin A (BioLegend; final concentration 0.08 µM phorbol-12-myristate 13-acetate (PMA) and 1.34 µM ionomycin) was added for the final 6 hours of culture to stimulate intracellular cytokine production. For each stimulated sample, an unstimulated condition containing no Cell Activation Cocktail was analyzed and used for setting the gating for cytokine-positive cells.

Post-culture, cells were washed twice and LIVE/DEAD Near-IR Fixable (Thermo Fisher Scientific) dye was added. Cells were then incubated with Human Fc block (BD Biosciences) prior to extracellular labeling as follows: anti-CD19-V500 (HIB19, BD Biosciences), anti-CD27-BV711 (O323, BioLegend), anti-IgD-BV421 (IA6-2, BioLegend), anti-CD38-BV785 (HIT2, BD Biosciences), anti-IgM-PE-Cy7 (MHM-88, BioLegend), anti-CD24-BUV395 (ML5, BD Biosciences), anti-CD3-APC-Cy7 (SKY7, BioLegend), and anti-CD5-AF700 (L17F12, BioLegend). Anti-PD-L1-FITC (MIH1, BD Biosciences) was also included in select experiments as indicated.

Cells were washed and fixed with BD Fixation and Permeabilization Solution (BD Biosciences). The cells were washed and intracellular labeling performed with anti-IL-10-AF647 (JES3-9D7, BioLegend), anti-TGF-β1-PE (TW4-9E7, BD Biosciences) and anti-TNF-α-AF488 (MAb11, BioLegend) in BD Perm/Wash buffer (BD Biosciences). Anti-IL-4-BV605 (MP4-25D2, BioLegend), anti-IFN-γ-AF700 (4S.B3, BioLegend) and anti-TLR9-PE (S16013D, BioLegend) were also included in select experiments. Cells were washed, acquired on the CytoFLEX Flow Cytometer (Beckman Coulter), and analyzed in FlowJo v10.4 (BD Biosciences). Gating strategies for the identification of live single CD19^+^ B cells, and TGF-β and PD-L1 isotype controls, are illustrated in Supplementary Figure 3(a-c). To account for the expansion in memory B cell lineages in cells, which received stimulation with 0.1 µg/ml CpG or CD40L + 10 µg/ml CpG (Supplementary Figure 4(a)), lineage analysis (identifying enriched phenotypes among cytokine^+^ B cells) was performed against the baseline phenotype of cytokine^−^ cells.

### B cell lineage and isotype profiling

CD19^+^ B cell lineage phenotypes were categorized according to CD24/CD38 expression: memory B cells (Bm; CD24^hi^ CD38^−^), transitional B cells (TrB; CD24^hi^ CD38^hi^), naïve B cells (CD24^int^ CD38^int^) and plasmablasts (PB; CD24^−^ CD38^++^). B cells were defined by antibody expression into non-class switched (IgM^+^IgD^+^, IgM^+^IgD^−^, IgM^−^IgD^+^) and class-switched IgM^−^IgD^−^ subsets.

### Dimensionality reduction using the tSNE and FlowSOM algorithms

Live single CD19^+^ B cells were gated, and dimensionality reduction was applied using the t-Distributed Stochastic Neighbor Embedding (tSNE) algorithm. Two-dimensional tSNE projections were generated utilizing the following parameters: CD27, IgD, IgM, CD24, and CD38. The Exact (vantage point tree) KNN and Barnes-Hut gradient algorithms with opt-SNE configuration (up to 1000 iterations) were used. The FlowSOM algorithm was used to generate six meta-clusters per sample based upon the five-marker panel.

### Single cell RNA-sequencing (scRNA-seq) analysis

Single-cell RNA-seq data from tumors of treatment-naive patients (N = 12, **Supplementary Table 2**) were selected from the publicly available dataset GSE123139.^21^ The data were analyzed using Seurat package (version 4.0.6).^22^ All cells (N = 19944) were filtered to include cells with gene counts greater than 200 and less than 3000. Data were normalized to 10000 counts per cell, log transformed, and 3000 highly variable features were identified (method = “vst”). Subsequently, data were scaled and principal component analysis performed. The first 30 PCs and a resolution were used to cluster the cells with the Louvain algorithm (resolution 1). DoubletFinder^23^ was used to remove the predicted doublets, and the remaining cells (N = 18325) were used for downstream analyses.

Clusters containing B cells (N = 2284, markers: *CD79A, CD79B, MS4A1*) and plasma cells (N = 268, markers: *CD38, SDC1*) were selected and reanalyzed in the same manner using 500 variable features, the first 10 PCs and a clustering resolution parameter of 0.5.

CellPhoneDB (version 2)^24^ was used to infer cell–cell interactions in the GSE123139 dataset after the DoubletFinder step. Clusters annotated as T cells (markers: *CD3E, CD4, CD8A*) and B cells (markers: *CD79A, CD79B, MS4A1*) were selected. T cells were split into conventional (Tcon, N = 10442) and regulatory T cells (Treg, N = 703, marker: *FOXP3*). B cells were subsetted into TGF-β^+^ (N = 269) or TNF-α^+^ (N = 22) based on non-zero counts of *TGFB1* and *TNF* genes in the raw data. Interactions were filtered using FDR < 0.001, and manually annotated into communication pathways associated with cell–cell contact, inhibitory checkpoints, co-stimulation, pro-inflammatory mediators, lymphocyte homing, recruitment, and assembly, leukotriene synthesis, negative regulation of inflammation, and inhibition of B cell responses.

### Bulk RNA-seq analysis

The melanoma dataset from TCGA-SCKM^25^ was separated based on tumor type (primary/metastatic) and site of resection or biopsy into primary (N = 103), metastatic skin (N = 116) and metastatic viscera (N = 36). TPM-normalized values were used to calculate Spearman’s rank correlation between selected genes using ggpubr R package (version 0.4.0). Additional analyses were performed using the UALCAN online tool.^26^

### Spatial transcriptomics analysis

Melanoma tumor samples frozen in OCT from three different patients were processed following 10x Spatial Transcriptomics protocol, with permeabilization time of 24 minutes and tissue thickness of 10µm. FASTQ files were mapped to human genome reference (GRCh38-2020-A), and the sequencing data was processed using SpaceRanger v1.3.1 (10x Genomics). Raw counts data were extracted in *Seurat*, and presence of at least one marker gene at non-zero counts was used to classify cell populations per spot: T cells (*CD3D, CD3E, CD3G*, CD247, and *CD4*), Tregs (*FOXP3, IL2RA, and TNFRSF4)*, and B cells (*CD19, MS4A1, CD79A, and CD79B*). Venn diagrams were created using R package *ggvenn* (v0.1.9).

### Cytokine suppression assay in B and T-helper cell co-culture

CD19^+^ B lymphocytes and CD4^+^ T-helper (Th) lymphocytes were isolated from peripheral blood of melanoma patients using the BD FACS Aria II cell sorter. Purified B and Th lymphocyte suspensions were co-cultured (1x10^5^ each/well) in sterile DMEM (10% FBS, 50 U/ml Pen-Strep) media containing Dynabeads® Human T-Activator CD3/CD28 (1x10^5^ beads/well; Thermo Fisher Scientific) and 10 U/ml recombinant IL-2 (PeproTech). 10 µg/ml CpG ODN 2006 was added to selected co-culture wells. Th cells (1x10^5^) were also cultured alone as control. 100 µl per well was added to round-bottom 96 well plates for each condition. The plates were incubated at 37°C with 5% CO2 for 72 hours. Cell Activation Cocktail (with Brefeldin A; BioLegend) was added for the final 6 hours of culture. For each sample, an unstimulated condition containing no Cell Activation Cocktail was analyzed.

Post-culture, cells were washed twice and LIVE/DEAD Near-IR Fixable dye was added. Cells were then incubated with Human Fc block (BD Biosciences) prior to extracellular labeling with anti-CD4-PE (A161A1, BioLegend). Cells were washed and fixed with BD Fixation and Permeabilization Solution (BD Biosciences). The cells were washed and intracellular labeling performed with anti-IFNγ-APC (4S.B3, BioLegend) and anti-TNF-α-AF488 (MAb11, BioLegend) in BD Perm/Wash buffer (BD Biosciences). Cells were washed, acquired on the CytoFLEX Flow Cytometer (Beckman Coulter) and analyzed in FlowJo v10.4 (BD Biosciences).

### Treg induction assay of B and conventional T-helper lymphocyte co-culture

CD19^+^ B lymphocytes and CD4^+^ CD25^−/int^ CD127^+^ conventional T-helper (Tcon) lymphocytes were isolated from peripheral blood of melanoma patients using the BD FACS Aria II cell sorter. Purified B and Tcon lymphocyte suspensions were co-cultured (1x10^5^ each/well) in sterile DMEM (10% FBS, 50U/ml Pen-Strep) media containing Dynabeads® Human T-Activator CD3/CD28 (1x10^5^ beads/well) and 10 U/ml recombinant IL-2. Tcon cells (1x10^5^) were also cultured alone as control. 100µl per well was added to round-bottom 96 well plates for each condition. The plates were incubated at 37°C with 5% CO2 for 72 hours.

Post-culture, cells were washed twice and LIVE/DEAD Near-IR Fixable dye was added. Cells were then incubated with Human Fc block (BD Biosciences) prior to extracellular labeling with anti-CD4-PE (A161A1, BioLegend). Cells were washed and fixed with Foxp3 Fixation/Permeabilization solution (Thermo Fisher Scientific). The cells were then washed in Permeabilization Solution (Thermo Fisher Scientific) and intracellular labeling performed with anti-FOXP3-AF488 antibody (259D, BioLegend). Cells were washed, acquired on the CytoFLEX Flow Cytometer (Beckman Coulter) and analyzed in FlowJo v10.4.

### Cellular proliferation assay of B and T-helper lymphocyte co-cultures

CD19^+^ B lymphocytes and CD4^+^ T-helper (Th) lymphocytes were isolated from peripheral blood of melanoma patients using RosetteSep™ Human B and CD4^+^ T Cell Enrichment Cocktails, respectively (STEMCELL Technologies). Purified B and Th lymphocyte suspensions were stained with 0.5µM eBioscience Cell Proliferation Dye eFluor 670 (Thermo Fisher Scientific) in PBS for 10 minutes at 37°C and washed three times in sterile ExCellerate B Cell Media (Bio-Techne) with 50U/ml Pen-Strep. B and T lymphocytes were co-cultured (1x10^5^ each/well) in media containing Dynabeads® Human T-Activator CD3/CD28 (1x10^5^ beads/well) and 10 U/ml recombinant IL-2. 50µg/ml Nivolumab (Bristol Myers Squibb) was added to selected co-culture wells. Th cells (1x10^5^) were also cultured alone as control. 0.01–1 µg/ml recombinant IL-10 or TNF-α (BioLegend) was added to selected T-helper monoculture wells. 100µl per well was added to round-bottom 96 well plates for each condition. The plates were incubated at 37°C with 5% CO_2_ for 72 hours.

Post-culture, cells were washed twice and LIVE/DEAD Near-IR Fixable dye was added. Cells were then incubated with Human Fc block (BD Biosciences) prior to extracellular labeling with anti-CD4-PE (A161A1, BioLegend). Cells were washed, acquired on the CytoFLEX Flow Cytometer (Beckman Coulter), and proliferation modeling was performed in FlowJo v10.4.

### Immunofluorescence staining of tumor specimens

Sections of frozen metastatic melanoma tumor tissue were thawed at room temperature (RT) and fixed with pre-cooled Acetone and Ethanol for 10 minutes each. Sections were washed and incubated with blocking buffer (0.1% BSA in 0.1% TBS-T) for 1 hour at RT, prior to primary antibody incubation for 1 hour at RT with one of either 5µg/ml rabbit anti-IL-10 (polyclonal, Abcam), 8µg/ml rabbit anti-TNF-α (TNFA/1500R, Abcam), or 1µg/ml rabbit anti-TGFβ1 (EPR21143, Abcam) in addition to 10µg/ml rat anti-CD3 (CD3-12, Abcam) and mouse anti-CD20 (1/50 dilution, L26, Abcam). Sections were washed and incubated with 10µg/ml cross-adsorbed donkey secondary antibodies (Abcam): anti-rabbit IgG AF594, anti-mouse IgG AF488 and anti-rat IgG AF647. Sections were washed and mounted with ProLong Gold Antifade Mountant with DAPI (Thermo Fisher Scientific). Slides were incubated for 24 hours prior to visualization on the Olympus VS120-S6-W slide scanning microscope.

### Statistical analyses

All data represent mean values ± standard error (SEM). Analysis of differences in mean between two distinct groups was performed using the student’s T-test for unpaired samples, and paired T-test for matched samples. All t-tests were two-tailed. Where multiple groups are compared, two-way Analysis of Variance (ANOVA) with Tukey’s multiple comparison test was employed. Survival analysis was performed using the log-rank (Mantel-Cox) test. P values were reported with the following associated symbols: P > .05 (ns), P < .05 (*), P < .01 (**), P < .001 (***), P < .0001 (****). P < .05 was considered to represent statistical significance.

## Results

### Enriched TGF-β^+^ and PD-L1^+^ regulatory and reduced TNF-α^+^ and IFN-γ^+^ pro-inflammatory B cell populations in melanoma patient circulation

**Figure 1.**
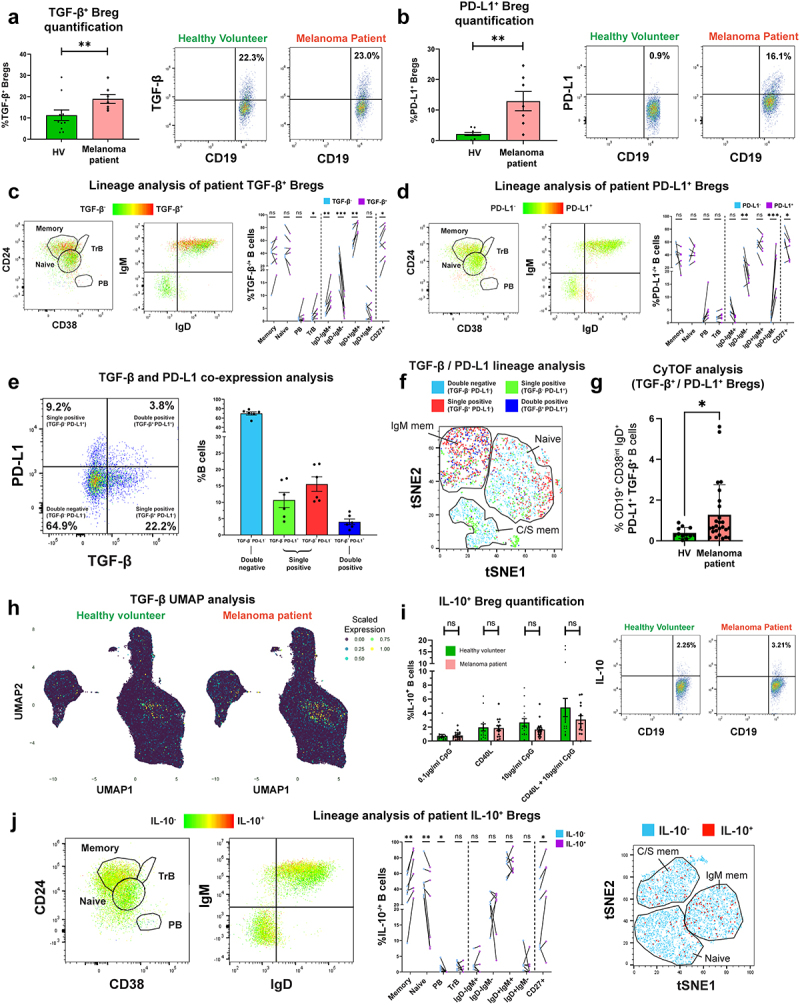
TGF-β- and PD-L1-expressing Bregs are enriched in melanoma patient compared to healthy volunteer peripheral blood and show preference toward specific B cell lineage phenotypes.

In circulating B cells of melanoma patient and age- and sex-matched healthy volunteer (HV) cohorts (characteristics in **Supplementary Table 1 & Supplementary Table 2)** we first explored expression the regulatory cytokines TGF-β and IL-10, using a flow cytometric intracellular cytokine assay. We found enrichment in TGF-β and PD-L1-expressing regulatory B cell (Breg) populations among the patient compared to the HV group (Figure 1(a-b)). Lineage analysis of both Breg compartments revealed that TGF-β-expressing B cells were significantly more likely to express CD27, IgD, and IgM compared to the baseline phenotype (TGF-β^−^ cells), denoting that TGF-β was associated with memory B cells of a non-isotype-switched phenotype (Figure 1(c)). Similarly, PD-L1-expressing B cells were significantly more likely to express IgD compared to the baseline, suggesting that PD-L1 expression may be associated with a non-isotype switched B cell phenotype (Figure 1(d)). In patient blood, single positive (TGF-β^−^ PD-L1^+^ and TGF-β^+^ PD-L1^−^) and double positive (TGF-β^+^ PD-L1^+^) B cell populations were present across B cell lineages with TGF-β^+^ PD-L1^+^ being prevalent among non-class switched (IgM^+^) B cells (Figure 1(e-f), Supplementary Figure 4(b)). CyTOF analyses of peripheral blood B cells (CyTOF 34-marker panel) identified enriched circulating TGF-β and PD-L1-expressing CD19^+^ CD38^int^ IgD^+^ CD27^−^ Bregs in melanoma patients (N = 26) compared to age- and sex-matched HV (N = 12) (Figure 1(g-h)). Contrastingly, detection of IL-10-expressing B cells using different *ex vivo* B cell stimuli showed no significant differences in the proportion of circulating IL-10^+^ cells in the CD19^+^ B cell compartment (Figure 1(i)), with lineage analysis showing expression in the memory phenotype (Figure 1(j), Supplementary Figure 4 (c)).

(a-b) [Left] Comparison of % TGF-β-expressing (a) and PD-L1-expressing (b) B cells in melanoma patients (N = 7) and matched healthy volunteers (N = 11). [Right] Flow cytometry plots show gating of CD19^+^ B cells by TGF-β or PD-L1 expression from a representative healthy volunteer and melanoma patient. (c-d) Live single CD19^+^ B cells from melanoma patient blood were gated prior to TGF-β^+^ (c) or PD-L1 (d) B cell lineage analyses. [Left] Flow cytometry plots highlighting identification of B cell lineage (CD24^hi^CD38^−^ memory B cells, CD24^hi^CD38^hi^ transitional B cells (TrB), CD24^int^CD38^int^ naïve B cells and CD24^−^CD38^++^ plasmablasts (PB)), and immunoglobulin isotype distribution in a representative patient. Cells are colored according to TGF-β or PD-L1 expression (gree low; red = high). [Right] B cell lineage analysis by TGF-β or PD-L1 expression (N = 7). % TGF-β- or PD-L1-expressing B cells showing particular B cell lineage phenotypes versus baseline phenotype (TGF-β-negative or PD-L1-negative cells) to identify phenotypic preference. (e) TGF-β/PD-L1 co-expression analysis showing single (TGF-β^−^ PD-L1^+^; TGF-β^+^ PD-L1^−^) and double (TGF-β^+^ PD-L1^+^) expressing populations. [Left] Flow cytometry dot plot for representative melanoma patient sample and [right] quantification for N = 6 melanoma patients. (f) tSNE projections cluster B cells according to CD27, IgD, IgM, CD24, and CD38 expression. Largest populations by FLOWSOM clustering were naïve, IgM memory, and isotype-switched (C/S) memory B cells. Cells were colored according to TGF-β and PD-L1 expression (light blue = double negative, green/red = single positive, dark blue = double positive). (g) CyTOF phenotyping of B cell subsets (34-marker panel) identified enrichment in CD19^+^CD38^int^IgD^+^CD27^−^ PD-L1^+^TGF-β^+^ regulatory B cells in patients (N = 26) compared to matched healthy volunteers (N = 12). (h) UMAP clustering analysis demonstrates enriched TGF-β^+^ (green and yellow) phenotypic population distribution in melanoma patients versus healthy volunteers. Cells clustered according to extracellular marker expression (CyTOF 19-marker panel) and colored corresponding to scaled expression of TGF-β (purple (0.00) to yellow (1.00)). (i) [Left] Comparisons of % IL-10-expressing B cells in melanoma patients (N = 18) and matched healthy volunteers (N = 17) following 72-hour culture with activation stimuli (0.1 µg/ml CpG, CD40L, 10 µg/ml CpG, or CD40L+10 µg/ml CpG). [Right] Flow cytometry plots show gating of CD19^+^ B cells by IL-10 expression from a representative healthy volunteer and melanoma patient (CD40L+10 µg/ml CpG condition). (j) B cell lineage analyses of live single IL-10^+^CD19^+^ B cells from melanoma patient blood. [Left] Flow cytometry plots highlighting B cell lineage and immunoglobulin isotype distribution in a representative patient (cells colored according to IL-10 expression, green = low; red = high). [Middle] Lineage analysis of B cells by IL-10 expression (N = 7). IL-10-expressing B cells were more likely to possess a memory B cell phenotype, above baseline. [Right] tSNE projections cluster B cells according to CD27, IgD, IgM, CD24, and CD38 expression by FLOWSOM clustering (cells colored according to IL-10 expression, light blue = double negative, red = positive). P values: P > .05 (ns), P < .05 (*), P < .01 (**), P < .001 (***).

**Figure 2.**
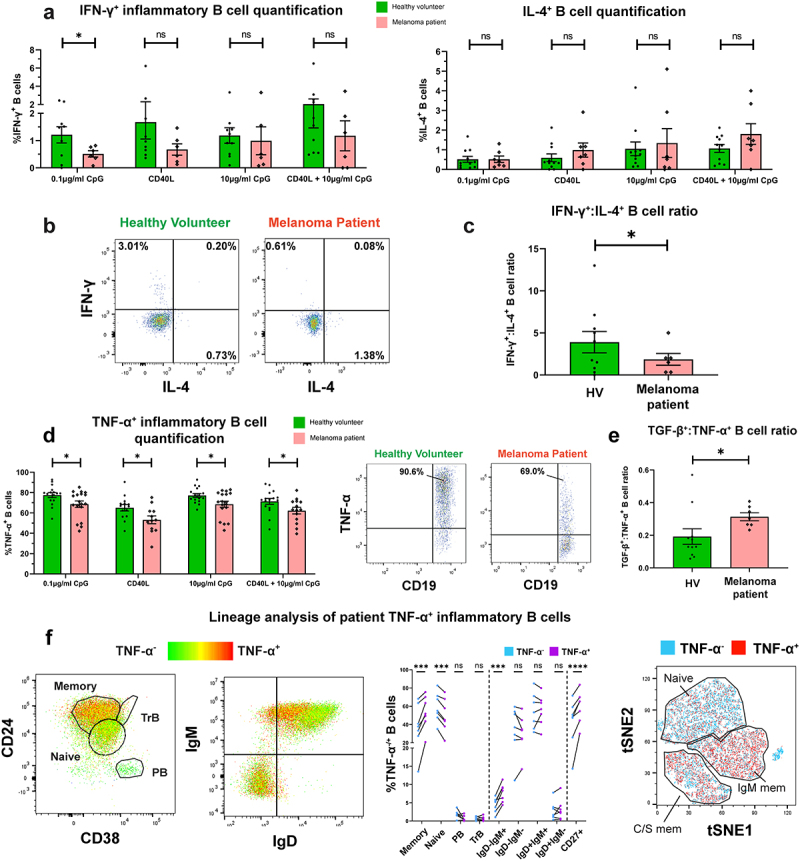
IFN-γ^+^ and TNF-α^+^ B cells are significantly downregulated in melanoma compared to healthy volunteer blood, and patient TNF-α^+^ B cells show preference for CD27^+^ memory B cell phenotypes.

We next evaluated circulating B cells expressing pro-inflammatory cytokines IFN-γ and TNF-α, and the Th2 cytokine IL-4. In the small IFN-γ^+^ B cell populations following innate stimulation (CpG ODN 2006), we observed significantly lower proportions of IFN-γ^+^ inflammatory B cells in melanoma patients compared to matched HV (Figure 2(a-b)). No significant differences were found in circulating IL-4-expressing CD19^+^ B cells (Figure 2(a-b)). However, the ratio of IFN-γ^+^:IL-4^+^ B cells, a pro-inflammatory measure, which may represent the balance between Th1 (IFN-γ^+^) and Th2 (IL-4^+^) phenotypes,^27^ was significantly lower in melanoma patients compared to matched HV (Figure 2(c)).

(a) Comparison of % IFN-γ-expressing [left] and IL-4-expressing [right] B cells in melanoma patients (N = 6/7 respectively) and matched healthy volunteers (N = 10/12 respectively) following 72-hour culture with B cell activation stimuli (0.1 µg/ml CpG, CD40L, 10 µg/ml CpG, or CD40L + 10 µg/ml CpG). (b) Flow cytometry plots of IFN-γ and IL-4 expression by CD19^+^ B cells from representative healthy volunteer and melanoma patient (0.1 µg/ml CpG). (c) Ratio of IFN-γ^+^:IL-4^+^ B cells in melanoma patients (N = 6) and matched healthy volunteers (N = 10) following stimulation with 0.1 µg/ml CpG. (d) [Left] Comparison of % TNF-α-expressing B cells in melanoma patients (N = 17) and matched healthy volunteers (N = 18) following 72-h culture with stimuli (0.1 µg/ml CpG, CD40L, 10 µg/ml CpG, or CD40L + 10 µg/ml CpG). [Right] Flow cytometry plots of TNF-α^+^CD19^+^ B cells from a representative healthy volunteer and melanoma patient (0.1 µg/ml CpG). (e) Ratio of TGF-β+:TNF-α^+^ B cells in melanoma patients (N = 7) and matched healthy volunteers (N = 11). TNF-α expression was detected following B cell activation with 0.1 µg/ml CpG. (f) TNF-α^+^ lineage analyses of live single CD19^+^ B cells from melanoma patient blood. [Left] Flow cytometry plots of B cell lineage and immunoglobulin isotype distribution in a representative patient (cells colored according to TNF-α expression: green = low; red = high). [Middle] Lineage analysis of B cells by TNF-α expression (N = 7). [Right] tSNE projections cluster B cells according to CD27, IgD, IgM, CD24, and CD38 expression using FLOWSOM clustering (cells colored according to TNF-α expression: light blue = double negative, red = positive). P values: P > .05 (ns), P < .05 (*), P < .001 (***), P < .0001 (****).

In contrast, a substantial proportion of patient and HV circulating B cells responded to innate pathway activation with polarized expression of TNF-α. However, we observed a collapse in the proportions of circulating TNF-α-expressing B cells in melanoma patients compared to matched HV (Figure 2(d)) evident across all B cell stimulation conditions. Moreover, the ratio of TGF-β^+^:TNF-α^+^ B cells was significantly increased in melanoma patients compared to matched HV, suggesting an overall skew toward regulatory cytokine expression in patient B cells (Figure 2(e)). TNF-α-expressing B cells were also significantly more likely to possess a memory B cell phenotype compared to the baseline (Figure 2(f), Supplementary Figure 4(d)).

These findings point to a level of dysregulation among melanoma patient circulating B cells, evident through expanded populations of TGF-β^+^, PD-L1^+^ Breg, and reduced IFN-γ^+^, TNF-α^+^ pro-inflammatory B cell compartments in the total peripheral blood B cell populations of patients with melanoma.

### TGF-β-expressing B cells infiltrate melanoma lesions, while TNF-α-expressing B cell populations are collapsed among tumor-infiltrating B lymphocytes (TIL-B)

**Figure 3.**
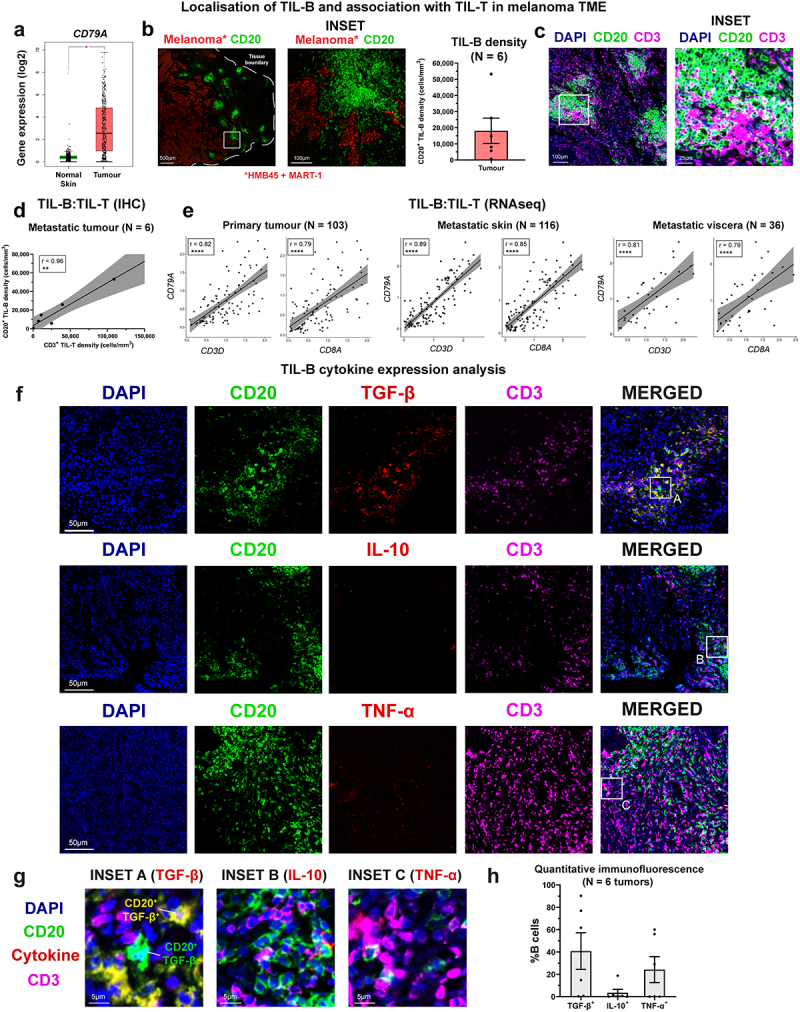
Prevalent TGF-β-expressing Bregs and rare TNF-α-expressing inflammatory B cells in melanoma lesions.

We next sought to investigate melanoma tumor-infiltrating B lymphocytes (TIL-B) and their cytokine expression profiles by immunohistochemistry, flow cytometric analyses, combined with bulk and single-cell RNA-seq analyses from publicly available datasets. Gene expression data from melanoma cutaneous lesion and normal skin cohorts (N = 1019 tissues) showed enhanced expression of the pan-B cell marker CD79A in melanoma compared to normal skin (Figure 3(a)) and immunohistochemical/immunofluorescence (IHC/IF) analyses showed the presence of TIL-B within clusters located adjacent to melanoma tumor islets (Figure 3(b)).

(a) [Left] Comparison of CD79A gene expression within N = 461 melanoma lesion samples and N = 558 normal skin tissues. The pan-B cell marker is significantly elevated in tumors. (b) [Left and middle] Example immunofluorescence images depicting the presence of CD20^+^ B cell clusters (green) adjacent to melanoma tumor islets (red). CD20; green, melanoma (HMB45 + MART-1); red. Scale bar = 500 µm (main image) and 100 µm (inset). [Right] Quantification of CD20^+^ TIL-B density in N = 6 melanoma tumors. (c) Example immunofluorescence images depicting clustering of CD20^+^ B cells (green) with CD3^+^ T cells within the melanoma TME. DAPI; blue, CD20; green, CD3; purple. Scale bar = 100 µm (main image) and 25 µm (inset). (d) CD20^+^ TIL-B and CD3^+^ TIL-T densities measured from N = 6 melanoma tumors show positive correlation (r = 0.96). (e) TIL-B (CD79A^+^) positively correlate with TIL-T (CD3D^+^), and TIL-cytotoxic T (CD8A^+^), analyzed by bulk RNA-seq gene expression (TCGA cohort) across cancer settings (primary, metastatic skin, and metastatic viscera). Spearman’s rank correlation test was used to calculate correlation coefficients (r) and p-values. (f-g) Example immunofluorescence images demonstrate strong presence of CD20^+^TGF-β^+^ Bregs (top row; yellow) within a melanoma tumor section, while limited presence of CD20^+^IL-10^+^ and TNF-α^+^ B cells was observed. DAPI; blue, CD20; green, TGF-β/IL-10/TNF-α; red, CD3; purple. Scale bar = 50 µm (panel images) and 10 µm (inset images). (h) Quantitative immunofluorescence (IF) analysis of cytokine expression among TIL-Bs from N = 6 tumor lesions. Percentages of TGF-β^+^, IL-10^+^, and TNF-α^+^ cells were measured among the B cell infiltrate. P values: P < .05 (*), P < .01 (**), P < .0001 (****).

IHC/IF evaluations also identified accompaniment of T cells within the TIL-B clusters in the melanoma TME, prominent in peritumoral areas (Figure 3(c)), and established a positive correlation between CD20^+^ TIL-B and CD3^+^ TIL-T densities among tumors (N = 6) (Figure 3(d)). Consistent with these observations, bulk RNA-seq gene expression data (TCGA cohort) analyses of human melanoma samples across primary lesions, metastatic skin, and visceral metastases, confirmed TIL-B (*CD79A*) gene expression to positively correlate with tumor-infiltrating T lymphocyte (TIL-T) (*CD3*), and cytotoxic T lymphocyte (*CD8A*^+^) gene expression, in both primary melanomas, as well as skin and visceral metastases (Spearman’s rank correlation test to calculate correlation coefficients (r) and p-values) (Figure 3(e)).

Although there were no significant differences in overall expression of TGF-β, IL-10, or TNF-α between melanoma lesions and normal skin (bulk RNA-seq, Supplementary Figure 5), IHC/IF evaluations identified cytokine-expressing TIL-B, found in close proximity to CD3^+^ TIL-T (Figure 3(f-g), Supplementary Figure 6). IHC/IF analyses of melanoma lesions identified substantial populations of TGF-β-expressing and TNF-α-expressing CD20^+^ TIL-B in melanoma lesions, while the presence of IL-10+ TIL-B was less frequent (Figure 3(h)). Additionally, intracellular cytokine phenotyping of TIL-B within single-cell suspensions obtained from melanoma lesions evaluated by flow cytometry showed no significant differences in the low percentages of IL-10-expressing Bregs among CD19 + B cells in the TME compared to the patient circulation, while TNF-α-expressing (CD19^+^) B cells were significantly reduced in melanoma lesions compared to the circulation (Supplementary Figure 7(a-c)).

Collectively, our results reveal cytokine-expressing TIL-B in melanoma, and evidence of clustering and association with T cells in the TME. These provide support for prominent regulatory TGF-β-expressing B cell profiles in melanoma lesions.

### Associations and crosstalk between TGF-β^+^ and TNF-α^+^ B cells with T cells in the tumor microenvironment (TME)

Based on our observations of cytokine expressing B cells found in clusters with T cells in melanoma lesions, we wished to investigate whether B cells, including cytokine-expressing Breg populations, may engage in functional crosstalk with the T cell response in melanoma.

**Figure 4.**
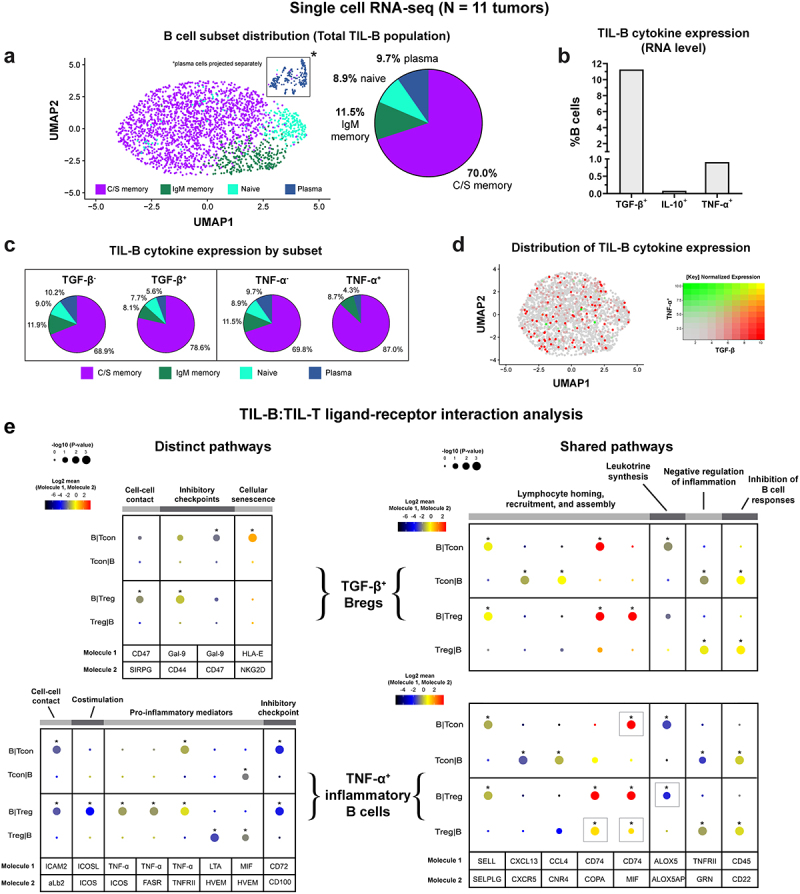
TGF-β and TNF-α-expressing B cells engage in functional crosstalk with Tcon and Treg cells in the TME.

In a single-cell RNA-seq analysis of pooled TIL-B from 12 samples of cutaneous melanoma lesions from a publicly available dataset (GSE123139)^21^ enriched in stage III individuals (Supplementary Table 2), we identified 2529 B cells, including a proportion of tumor-infiltrating TGF-β^+^ Bregs, (11.3%), while populations of IL-10^+^ (0.1%) and TNF-α^+^ (0.9%) TIL-B were less frequently detected (Figure 4(a-b)), in agreement with our quantitative IHC/IF analyses (Figure 3(h)). Both tumor-infiltrating TGF-β^+^ (78.6%) and TNF-α^+^ (87.0%) B cells were predominantly of the class-switched memory phenotype (Figure 4(c)), supporting the notion that memory B cells may be a key source of cytokine expression in melanoma lesions. In addition, minimal TGF-β and TNF-α co-expression was observed among TIL-B (Figure 4(d)), suggesting that the expression of each cytokine may be mutually exclusive among the B cell tumor infiltrate.

(a) [Left] UMAP visualization defined by global GEx of single melanoma TIL-B pooled from N = 11 patients (2529 cells), highlighting distribution of major B cell subsets (C/S memory, IgM memory, naïve, and plasma cells). [Right] Pie chart illustrates the proportions of major B cell subsets among melanoma TIL-B. (b) Proportions of melanoma TIL-B expressing TGF-β, IL-10, and TNF-α. (c) Lineage analysis of melanoma TIL-B by cytokine expression. Pie charts compare lineage phenotypes of B cells expressing each of TGF-β [left panel], and TNF-α [right panels], to baseline phenotype (denoted as cytokine^−^ cells) to identify phenotypic preference. TGF-β and TNF-α-expressing TIL-B show a preference toward class-switched memory phenotypes. (d) UMAP visualization of melanoma TIL-B, colored by relative normalized gene expression levels for TGF-β and TNF-α. (e) CellPhoneDB analysis of TGF-β^+^ [top] and TNF-α^+^ [bottom] B cell:T cell communication pathways in the TME. T cells were divided into conventional (Tcon) and regulatory (Treg) subsets. [Left] TGF-β^+^ and TNF-α^+^ B cell subsets were found to engage in distinct cell–cell contact and inhibitory checkpoint interactions with T cells, while TNF-α^+^ B cells also engaged in co-stimulation and TNF-α signaling via ICOS, FasR, and TNFRII. [Right] Shared interactions (identical ligand:receptor pairs) for both B cell subsets include lymphoid homing, recruitment, and assembly, leukotriene synthesis, negative regulation of inflammation, and inhibition of B cell responses. Square gray boxes indicate where the shared pathway interactions differ between TGF-β^+^ and TNF-α^+^ B cell subsets. Significance was detected after the false discovery rate (FDR<0.001) correction. Circle sizes indicate that p-values and color-coding represents the average expression level of interacting molecule 1 in cluster 1 and interacting molecule 2 in cluster 2.

We then probed for signs of cell–cell interactions between cytokine-expressing B cells with conventional (Tcon) and regulatory (Treg) populations using CellPhoneDB (single-cell RNA-seq dataset^21^). Distinct B cell:T cell communication pathways were identified for TIL-B stratified by cytokine expression.^24^ While cell–cell contact, and inhibitory checkpoint interactions with conventional T cells and Treg cells were detected for both TGF-β^+^ and TNF-α^+^ B cells, the underlying ligand-receptor pairs differed (Figure 4(e)). For TGF-β^+^ B cells with Tregs, the cell-cell contact was supported by CD47:SIRP-γ.^28^ ICAM2:aLb2 interactions were shown for TNF-α^+^ B cells engaged with Tcon and with Treg populations.^29^ In addition, TGF-β^+^ B cells expressed the immune checkpoint receptor Galectin-9,^30^ which bound CD44 on Tregs, and CD47 on Tcon cells. Importantly, the interaction of Galectin-9 and CD44 was previously shown to promote FOXP3 expression and enhance the function and stability of induced Tregs (iTregs) in mouse models, alongside complex formation with TGF-β receptor I.^31^ TGF-β^+^ B cells also expressed HLA-E, a non-classical HLA molecule, which is upregulated in stressed and senescent cells,^32,33^ and interacted with NKG2D expressed by Tcon cells.

As expected, TNF-α^+^ B cells engaged in signaling with T cells via pro-inflammatory mediators, including TNF-α, lymphotoxin-α (LT-α), and macrophage migration inhibitory factor (MIF). Our analysis indicated that TNF-α expressed by B cells interacted with TNFRII on both Treg and Tcon cells, and TNF-α also signaled via FasR and ICOS expressed by Tregs. In addition, TNF-α^+^ B cells expressed the costimulatory ICOS ligand, which interacted with its receptor ICOS expressed by Tregs, and in this context may promote the generation, proliferation, survival, and suppressive ability of Tregs.^34,35^ Lastly, TNF-α^+^ B cells expressed CD72, a negative checkpoint regulator of B cell responsiveness,^36^ which interacted with CD100 expressed by both Tcon and Treg cells (Figure 4(e)). CellPhoneDB analyses also identified shared communication pathways, with common ligand-receptor pairs, which were present in B cell:T cell interactions involving either TGF-β^+^ or TNF-α^+^ B cells (Figure 4(e)). These shared interactions included molecules associated with lymphocyte homing, recruitment, and assembly (SELL, CXCL13, CCL4, and CD74), leukotriene synthesis (ALOX5), negative regulation of inflammation (GRN), and inhibition of B cell responses (CD22).

In summary, B cells in melanoma lesions are polarized to express regulatory cytokines, including TGF-β, and may engage in immunosuppressive crosstalk with tumor-associated T cells via Galectin-9 signaling. We found reduced tumor-resident TNF-α^+^ B cell populations compared to matched blood, and TNF-α^+^ TIL-B engaging in extensive crosstalk with Tregs, likely supporting immune cell suppressive activities in the TME.

### *Melanoma patient B cells support the proliferation of autologous T-helper cells and promote TGF-β-mediated differentiation of FOXP3^+^ Tregs* ex vivo

Since we detected crosstalk between cytokine-expressing B cells with T cells including Tregs, we investigated the influence of melanoma patient-derived B cells on autologous T cell phenotype and effector function in *ex vivo* B and T-helper cell co-culture studies.

**Figure 5.**
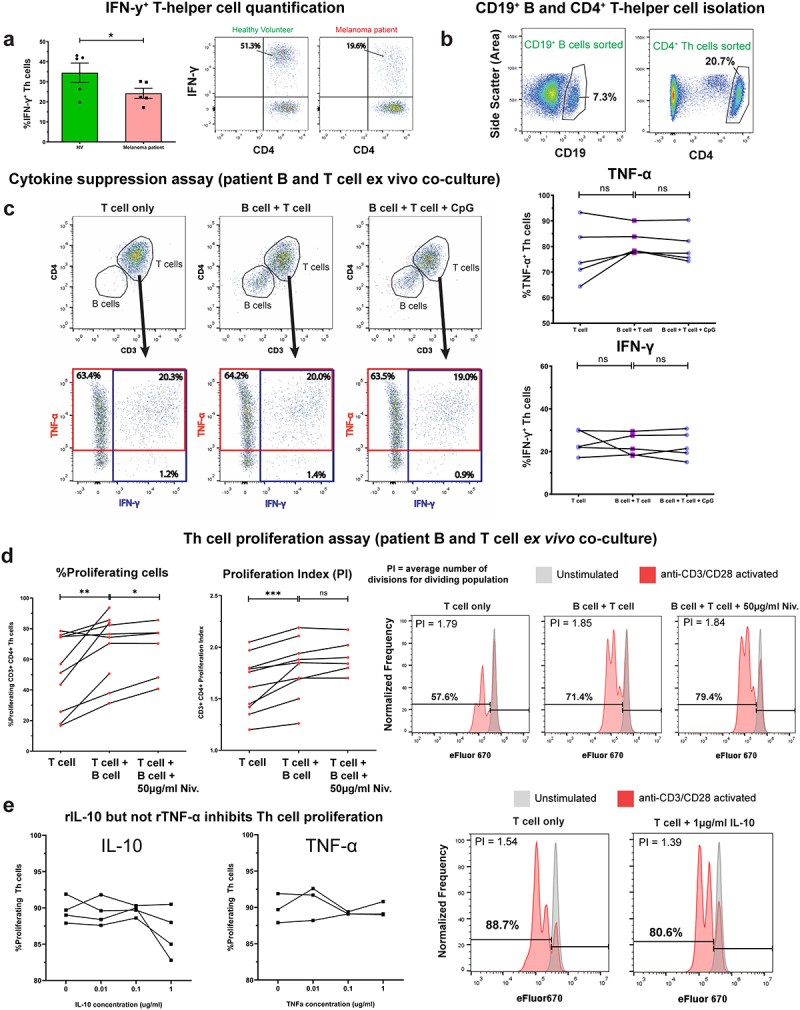
Patient-derived B cells did not suppress pro-inflammatory (IFN-γ and TNF-α) cytokine expression, and enhanced proliferation of autologous T-helper cells.

Consistent with our observation of reduced TNF-α^+^ inflammatory B cells in patient blood and tumors (Figure 2(d) and Supplementary Figure 7(a-c)), circulating pro-inflammatory IFN-γ-expressing CD4^+^ T-helper cells were significantly lower in melanoma patients compared to HV (Figure 5(a) in agreement with previous reports.^10^ In autologous B-T cell 1:1 co-cultures, melanoma patient-derived B cells did not suppress either IFN-γ or TNF-α-expression by CD4^+^ T-helper cells (Figure 5(b-c)). In contrast, patient B cells significantly enhanced both the proliferation index and the percentage of proliferating T-helper cells (Figure 5(d)). This pro-proliferative effect was further enhanced by PD-1/PD-L1 checkpoint blockade, although only through increases in the percentage of proliferating T-helper cells, and not the proliferation index. Recombinant TNF-α did not significantly modulate T-helper cell proliferation, while, as expected, recombinant IL-10 was found to inhibit proliferation^37^ (Figure 5(e)).

(a) The proportions of circulating IFN-γ^+^ T cells were significantly reduced in melanoma patients (N = 5) compared to healthy volunteers (N = 5). (b) Cell-sorting strategy for the isolation of CD19^+^ B and CD4^+^ T-helper cells from PBMC. (c) [Left] Flow cytometry plots indicate % T cells expressing IFN-γ and/or TNF-α from T-helper cells in “T cell only”, “B cell + T cell” and “B cell + T cell + CpG (ODN 2006)” culture conditions for a representative patient. [Right] Peripheral blood B cells isolated from five melanoma patients did not suppress IFN-γ and TNF-α expression by autologous CD4^+^ T-helper cells following ex vivo co-culture. (d) B cells from melanoma patients (N = 10) enhanced proliferation of autologous CD4^+^ T-helper cells. [Left] Addition of B cells into ex vivo T cell cultures enhanced both the % proliferating T-helper cells and the proliferation index (total number of divisions divided by the number of cells that went into division). Addition of 50 µg/ml the anti-PD-1 antibody nivolumab further boosted the % proliferating T-helper cells in co-cultures. [Right] Flow cytometry dot plots show generational peaks and indicate % proliferating T-helper cells in “T cell only”, “B cell + T cell”, and “B cell + T cell + 50 µg/ml nivolumab” conditions for a representative patient. (e) Recombinant IL-10 (1 µg/ml) can significantly inhibit the proliferation of melanoma patient peripheral blood-derived CD4^+^ T cells (N = 4) following anti-CD3/CD28 activation + IL-2, while recombinant TNF-α (0.01–1 µg/ml) had no significant effect (N = 3). [Right] Flow cytometry plots show generational peaks and indicate % proliferating T-helper cells in “T cell only” vs “T cell + 1 µg/ml IL-10” for a representative patient. P values: P > .05 (ns), P < .05 (*), P < .01 (**), P < .001 (***).

**Figure 6.**
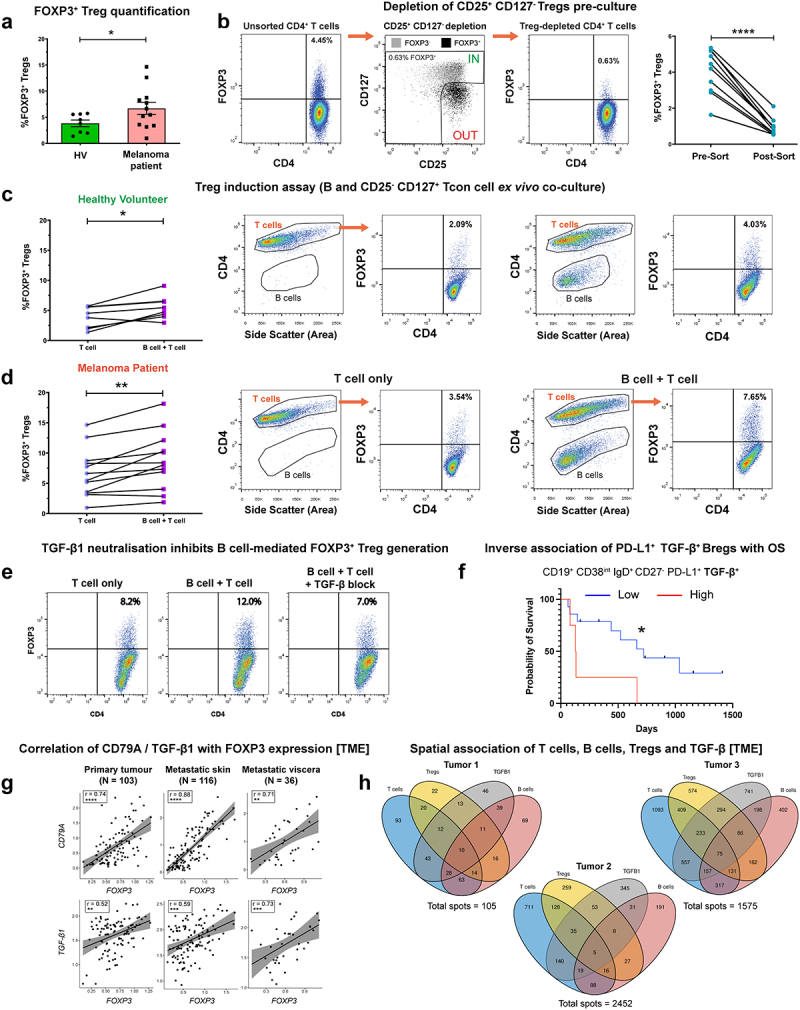
B cells derived from melanoma patient peripheral blood promote a FOXP3^+^ Treg phenotype from CD25^−/int^ CD127^+^ conventional T cells.

We examined melanoma patient-derived B cells in B:T-helper cell *ex vivo* co-cultures to evaluate whether these supported induction of FOXP3^+^ CD4^+^ regulatory T cells (Tregs). Initially, we observed a significantly increased percentage of FOXP3^+^ Tregs in melanoma patient compared to healthy volunteer peripheral blood, following anti-CD3/CD28 activation (Figure 6(a)). This suggested that a patient-specific subpopulation of T-helper cells may respond to activation by differentiating into a Treg phenotype. For the co-culture study, we purified populations of non-Treg (conventional) T cells by removing the CD25^+^ CD127^−^ Treg subset, which are enriched in FOXP3 expression (Figure 6(b)). E*x vivo* B:T cell co-cultures demonstrated that melanoma patient and healthy volunteer B cells significantly promoted FOXP3^+^ Treg differentiation from autologous purified CD25^−/int^ CD127^+^ conventional T-helper cells (Figure 6(c-d)). This suppressive effect was blocked when TGF-β1 was neutralized in cell cultures (Figure 6(e)), suggesting a link between TGF-β expression and induction of Tregs.

(a) The % of FOXP3^+^ Tregs are elevated in melanoma patients (N = 12) compared to healthy volunteers (N = 8), following anti-CD3/CD28 stimulation. (b) [Left] Cell-sorting strategy for the depletion of CD25^+^CD127^−^ Tregs from peripheral blood CD4^+^ T cells. [Right] FOXP3-expressing Tregs are successfully depleted following cell sorting (N = 10). (c-d) B cells isolated from healthy volunteers (N = 8) (c) and melanoma patients (N = 12) (d) peripheral blood promoted FOXP3^+^ Treg differentiation from CD4^+^CD25^−/int^CD127^+^ cells following *ex vivo* co-culture. Representative flow cytometry plots indicate % of FOXP3-expressing T-helper cells in “T cell only” vs “B cell + T cell” conditions. (e) Representative flow cytometry plots showing that antibody-mediated neutralization of TGF-β alleviates B cell-facilitated induction of FOXP3^+^ Tregs from CD4^+^CD25^−/int^CD127^+^ Tcon cells. (f) The presence of peripheral blood CD19^+^CD38^int^IgD^+^CD27^−^PD-L1^+^TGF-β^+^ naïve Bregs is associated with decreased probability of overall survival. Patients (N = 18) split by %PD-L1^+^TGF-β^+^ naïve Bregs into high (top quartile) and low (below top quartile) groups. (g) CD79A [top] and TGF-β [bottom] gene expression positively correlate with FOXP3, analyzed by bulk RNA-seq gene expression (TCGA cohort) across cancer settings (primary, metastatic skin, and metastatic viscera). Spearman’s rank correlation test was used to calculate correlation coefficients (r) and p-values. (h) Spatial transcriptomics analysis identifies the colocalization of T cells, B cells, Tregs, and TGF-β1 in three melanoma tumor specimens. 9.5% (tumor 1; 10/105), 0.2% (tumor 2; 5/2452), and 4.8% (tumor 3; 75/1575) of spots showed T cells, Tregs, B cells, and presence of TGF-β1. P values: P < .05 (*), P < .01 (**), P < .001 (***) P < .0001 (****).

In concordance with regulatory properties of TGF-β^+^ Bregs, Kaplan–Meier survival analysis of our melanoma patient cohort indicated that higher levels of circulating TGF-β-expressing CD19^+^ CD38^int^ IgD^+^ CD27^−^ PD-L1^+^ naïve B cells were associated with less favorable overall survival (N = 18) (Figure 6(f)). Within the melanoma TME, we found a positive correlation between CD79A with FOXP3 and between TGF-β with FOXP3, gene expression (Figure 6(g)). Moreover, we found FOXP3 expression to be preferentially expressed among the tumor-infiltrating T cell population, alongside frequent co-expression with TIGIT (Supplementary Figure 8(a-e)), confirming FOXP3 as a robust marker for Treg detection in the TME. Finally, spatial transcriptomics analysis of human melanoma lesions confirmed the localized presence of T cells, Tregs, B cells, and TGF-β1 within melanoma tumor specimens obtained from N = 3 individuals (Figure 6(h)).

Together, these findings highlight that melanoma patient B cells support the proliferation of autologous T-helper cells and promote autologous Treg differentiation through the expression of TGF-β. Elevated circulating naïve TGF-β^+^ B cells may indicate unfavorable survival outcomes in patients with melanoma.

## Discussion

The roles of B cells in cutaneous immune surveillance have received growing attention in recent years,^38^ with research demonstrating that populations of mature, isotype-switched B cells reside in healthy skin^39^ and that B cells may accumulate and proliferate in the skin in response to cutaneous antigenic challenge.^40^ Evidence has also been gathered for skin tumor-resident mature B cell and antibody compartments in human melanoma, which may generally confer positive prognostic value.^41^ Previous investigations have pointed to skewed isotype expression by B cells in melanoma, away from the prevalent IgG1, favoring B cells expressing regulatory isotypes such as IgG4 and IgA,^42,43^ which may suppress anti-tumor immune responses. It is also possible that cytokines including IL-4, IL-10, TGF-β, and VEGF, possibly combined with a corresponding reduction or impairment of pro-inflammatory cytokines such as IFN-γ, which would otherwise support isotype-switching to IgG1, may be part of the regulatory profile of a skewed humoral immune response in melanoma. In addition to tumor cells^44^ and T cells,^45^ B cells^46^ may be a source of these cytokines and may also act in a paracrine manner to influence T cell phenotype and functions. However, despite evidence suggesting that cytokine-expressing B cell subsets can support immune modulation and tumor progression in murine models, via the secretion of IL-10,^9^ the role of circulating and intratumoral cytokine-expressing B cells in patients with melanoma has received insufficient attention. We therefore sought to investigate the phenotype and functions of the cytokine-secreting B cell compartment, with focus upon unraveling regulatory B cell (including IL-10, TGF-β, and PD-L1-expressing subsets), and pro-inflammatory B cell (including IFN-γ and TNF-α-expressing subsets) profiles in human melanoma.

To provide a broad understanding of the potential dysregulation among cytokine-expressing B cells in patients with melanoma, we quantified regulatory and inflammatory B cell populations in cohorts of melanoma patients and matched healthy volunteers. We identified enhanced levels of regulatory B cells expressing either TGF-β or PD-L1 in melanoma patient compared to healthy volunteer peripheral blood. Consistently, a 34-marker CyTOF panel revealed enrichment in TGF-β^+^ CD19^+^ CD38^int^ IgD^+^ CD27^−^ PD-L1^+^ Bregs in patient compared to healthy volunteer circulation. We identified a concurrent collapse of the inflammatory B cell compartment, with significantly lower proportions of circulating IFN-γ^+^ and TNF-α^+^ inflammatory B cells in the patient compared to healthy volunteer circulation. Collectively, our analyses suggest an overall dysregulated cytokine-expressing B cell compartment in melanoma patient circulation, which appears to favor the induction of regulatory B cell (TGF-β^+^ and PD-L1^+^) subpopulations, alongside a collapse in pro-inflammatory (IFN-γ^+^ and TNF-α^+^) B cell subsets.

We also aimed to gain an insight into the cytokine-expressing cells categorized by B cell lineage subpopulations in the melanoma patient circulation, for which phenotypic descriptions of regulatory and inflammatory B cell subsets have not yet been determined. Our evaluations revealed that melanoma patient B cells expressing each of IL-10, TGF-β and TNF-α cytokines were present across B cell lineages, although with a marked preference toward a CD27^+^ memory phenotype, while TGF-β^+^ and PD-L1^+^ B cells were mostly found among non-isotype switched B cell subsets. To our knowledge, we provide the first report of the circulating memory B cell pool as an important source of both regulatory and pro-inflammatory cytokines in patients with melanoma. Future studies can explore the plasmablast and memory B cell pools as potential sources of cytokine-expressing cells.

Following our observations of systemic dysregulation among cytokine-expressing B cells in patients with melanoma, we sought to investigate cytokine expression profiles (regulatory IL-10 and TGF-β, and pro-inflammatory TNF-α) within the TIL-B compartment. In line with previous reports,^39,47^ we found enhanced B cell marker gene expression within melanoma lesions compared to normal skin. Our IHC/IF observations confirmed the presence of CD20^+^ TIL-B clusters, typically spatially associated alongside T cells, with significant cluster formation localized adjacent to melanoma tumor islets. The presence of B-T clusters in the tumor microenvironment has been reported in other cancer types,^48–50^ may identify areas of localized lymphoid assembly, activation, and differentiation, and are often characterized as tertiary lymphoid structures. The presence of these structures has also been shown to associate with response to therapies.^51,52^ Our observation of B-T clustering was consistent with our finding of TIL-B:TIL-T crosstalk (CellPhoneDB analyses of scRNA-seq immune cell signatures) via molecules such as SELL, CXCL13, CCL4, CD74 known to be involved in lymphocyte recruitment and assembly.

We found lower proportions of TNF-α expressed B cells in patient tumors compared to patient peripheral blood (which were already lower than those found in the circulation of healthy volunteers) using intracellular cytokine phenotyping. However, TNF-α^+^ CD20^+^ TIL-B were evident in human melanoma lesions by IHC/IF cytokine co-staining, and their presence was confirmed in an independent melanoma tissue cohort by scRNA-seq analyses. Lower levels of circulating TNF-α^+^ expressing B cells in patients compared to HV, and TNF-α^+^ expressing B cells proportionally less highly represented in the B cell TME compartment compared to patient blood may denote a degree of regulation of the pro-inflammatory humoral immune compartment in melanoma. The contributions of known pro-inflammatory cytokines such as TNF-α to anti-tumor immunity remain controversial. For example, TNF-α expression has been shown to mediate tumor cell apoptosis and promote immune cell proliferation and survival.^53^ In contrast, sustained inflammation is a hallmark of cancer,^54^ and it is possible that the associated chronic exposure to TNF-α and its network of ligands and receptors, including activation of the NF-κB pathway,^55^ can stimulate survival factors such as anti-apoptotic proteins.^56^ The latter may explain the previously observed inverse correlation between proportions of circulating TNF-α^+^ inflammatory B cells and response to checkpoint blockade in a cohort of melanoma patients.^12^

On the other hand, our data suggest that TGF-β-expressing Breg populations were prominent in patient blood, and both IHC/IF and scRNA-seq analyses in tumor lesions demonstrated the presence of a significant population of TGF-β^+^ B cells in the TME. Consistent with these cells likely supporting an immunosuppressive environment, *ex vivo* co-cultures of melanoma patient-derived B cells with autologous Tcon cells depleted of Tregs resulted in TGF-β-mediated induction of FOXP3^+^ Tregs. Although statistical significance was achieved for Treg induction by both healthy volunteer and melanoma patient B cells, the overall polarization of FOXP3^+^ Tregs following the co-cultures was most prominent within the melanoma patient cohort.

In the TME, TGF-β^+^ B:T cell spatial clustering and bidirectional communication by CellPhoneDB analyses pointed to modulatory signals evident from leukotriene synthesis (ALOX5),^57^ negative regulation of inflammation (GRN, encoding for progranulin)^58^ and inhibition of B cell responses (CD22).^59^ TGF-β^+^ B cells expressed the immune checkpoint receptor Galectin-9,^30^ which signaled with Tregs via CD44. Moreover, this interaction has been previously described to act synergistically with TGF-β signaling, to promote FOXP3 expression and to enhance the function and stability of induced Tregs.^31^ Together, our findings point to TGF-β regulatory Breg populations as potential players in interacting with T cells via a range of regulatory signals and functionally supporting the generation of Tregs. These observations represent previously undescribed findings, as the significance of melanoma patient B cells in contributing to Treg generation *ex vivo* has not been investigated before. Future studies may further unravel the regulatory network involving TGF-β, FOXP3, and Galectin-9,^60^ and aid our understanding of their synergistic significance in immune responses to solid tumors.

Our CellPhoneDB analyses also identified interactions between TNF-α^+^ TIL-B and Tregs, including via pro-inflammatory mediators TNF-α, LT-α, and MIF, and the ICOS/ICOSL axis, which may also drive the phenotypic stability and suppressive activity of Tregs.^34,35,61^ Together with our observations of TGF-β^+^ B cell/Treg crosstalk, these analyses reveal previously unknown interactions between cytokine-expressing B cell subsets and Tregs. Collectively, these observations highlight multiple immunoregulatory interactions between TGF-β^+^ and TNF-α^+^ TIL-B, with T cells in the TME. TGF-β^+^ B cells have senescent properties and may support the differentiation and maintenance of Tregs in melanoma lesions through expression of Galectin-9, while TNF-α^+^ B cells may engage in extensive crosstalk with Tregs, including via ICOS, TNF-α, LT-α, and MIF signaling, which are likely to support the suppressive activity of Tregs in the melanoma TME. Further investigations elucidating these interactions, including the potential contributions of the B cell:Treg ICOS/ICOSL axis, may reveal previously unappreciated players in immune responses to solid tumors.

On the other hand, we observed that B cells, including those extracted from patients with melanoma, may exert a positive influence on T cells. Strikingly, we found that patient-derived B cells promoted the *ex vivo* proliferation of autologous T-helper cells, while allowing autologous T-helper cells to produce pro-inflammatory cytokines (IFN-γ and TNF-α). These observations suggest that, despite the observed declines in pro-inflammatory cytokine expression, circulating B cells in patients with melanoma maintain their immunostimulatory capacities *ex vivo*. Interestingly, anti-PD-1 treatment further enhanced B cell-associated proliferative signaling on T cells, suggesting that B cell functions and interactions with T cells may be influenced by checkpoint inhibitors.

**Figure 7.**
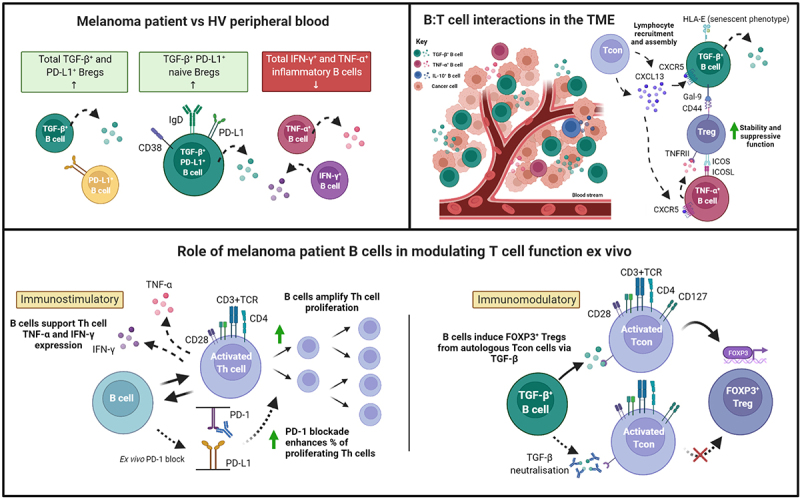
Cytokine-expressing B cells in melanoma are dysregulated in the circulation, engage in crosstalk with Tregs in the TME, and engender immunostimulatory and immunomodulatory influences ex vivo.

Together, our findings, illustrated in Figure 7, highlight the wider dichotomy of immune responses which appears to also be reflected in the phenotypes and functions of cytokine-producing B cell compartments in melanoma. The overall contributions of B cells toward tumor surveillance may hinge upon a balance between immunostimulatory and immunomodulatory capabilities, to which their expressed cytokines may play a role. Further investigations into the factors influencing this balance are warranted, especially in large cohorts of patients across disease stages and treatment groups, and therapies that compel B cell responses toward pro-inflammatory and immunostimulatory responses may offer new options for treatment success in the clinic.

Our results identified dysregulation among cytokine-expressing B cells in melanoma patient circulating B cell compartment which favored regulatory phenotypes. We found enriched circulating populations of total TGF-β, total PD-L1 regulatory B cells, and a TGF-β and PD-L1 co-expressingCD19^+^CD38^int^IgD^+^CD27^−^ naïve Breg subset, simultaneous to reduced pro-inflammatoryIFN-γ^+^ and TNF-α^+^ B cells in melanoma patients compared to matched healthy volunteers. Within melanoma lesions, TNF-α-expressing B cells were detectable but proportionally reduced compared to those present in the patient circulation, and substantial populations of intratumoral TGF-β^+^ B cells were detected. TGF-β^+^ B cells featured a HLA-E^+^ senescent phenotype and may support the differentiation and maintenance of Tregs in melanoma tumors through expression of Galectin-9. On the other hand, the TNF-α^+^ TIL-B engaged in crosstalk with Tregs, including via the ICOS/ICOSL axis and TNF-α signaling, which are likely to support the suppressive activity of Tregs in the melanoma TME. Expression of CXCL13 by Tcon cells may participate in the recruitment of both TGF-β^+^ and TNF-α^+^ B cells. Finally, ex vivo B:T cell co-cultures demonstrated that patient-derived B cells promoted the proliferation of autologous T-helper cells, which could be further enhanced with anti-PD-1 treatment, and also permitted autologous T-helper cells to produce pro-inflammatory cytokines (IFN-γ and TNF-α). In contrast, melanoma patient B cells significantly promoted ex vivo FOXP3^+^ Treg differentiation from autologous purified CD25^−/int^CD127^+^ conventional T-helper cells via TGF-β. Overall, we have established that cytokine-expressing B cells are dysregulated in the circulation of melanoma patients, can infiltrate tumors and engage in crosstalk with and promote Tregs in the TME, while still being able to participate in immunostimulatory T cell functions ex vivo.

## Supplementary Material

Supplemental Material

## Data Availability

The data that support the findings of this study are available from the corresponding author (S.N.K.) upon reasonable request. The data are not publicly available due to containing information that could compromise the privacy of research participants.
