# Supplementary material for: Enriched circulating and tumor-resident TGF-β+ regulatory B cells in patients with melanoma promote FOXP3+ Tregs
Source: Oncoimmunology. 2022 Jul 28;11(1):2104426. doi: 10.1080/2162402X.2022.2104426 (PMC9336482; doi:10.1080/2162402X.2022.2104426)
Supplement: Supplemental Material [file KONI_A_2104426_SM9602.zip › Supplementary Figure Captions.docx]

# Supplementary Materials and Methods

## Cell staining for mass cytometry

CyTOF staining was performed on frozen PBMCs. After thawing cells were counted with the aim of staining 2-4 x 10^6^ per tube, where cell yield was higher the samples were split into 2. Thawed PBMCs were added to filter-cap FACS tubes and washed with 3ml of MaxPar Cell Staining Buffer (Fluidigm) at 800xg for 5 minutes. Cell pellets were re-suspended (volume 80μl) and added to a second FACS tube. Cells were incubated with 5µl of Fc Blocking Solution (Human TruStain Fcx^TM^ (Fc Receptor blocking solution) (Biolegend)) for 10 minutes at room temperature. A cell surface staining antibody cocktail of 31 antibodies including pre-made antibodies (Fluidigm) as well as in-house conjugated antibodies was added and incubated with the cells for 30 minutes on ice. All antibodies were vortexed and spun at 10000g for 5 minutes prior to adding to prevent possible antibody aggregates. During the last 1 minute of incubation, 194 Cisplatin (Fluidigm) live/dead stain was added at a concentration of 5μM for 2 minutes. Initial titration of live/dead staining with healthy volunteer samples (N=2) revealed overstaining; therefore, for subsequent experiments the time was reduced to 1 minute. The cells were quenched with 4ml of MaxPar cell staining buffer and washed at 800g for 5 minutes. The cells were then fixed using Fix I buffer (Fluidigm) at room temperature for 15 minutes. The fixed cells were washed twice in Perm S permeabilisation buffer (Fluidigm). Intracellular staining cocktail, also consisting of Fluidigm pre-conjugated as well as in-house conjugated antibodies was prepared and added to the cells for 30 minutes at room temperature. After washing with MaxPar cell staining buffer cells were incubated overnight at 4°C with intercalation solution consisting of DNA intercalator 103-Ir (Fluidigm) and Fix + Perm Buffer (Fluidigm). Finally, after overnight incubation cells were washed with MaxPar Cell staining Buffer (Fluidigm) and twice in MilliQ water. EQ Calibration beads were added as per the manufacturer’s directions prior to running. Data were acquired on a Helios mass cytometer (Fluidigm) in batches.

## Quantification and data analysis for mass cytometry

Data were obtained in the form of .fcs files from the Helios machine. Files were normalised and concatenated using Fluidigm CyTOF Software. Pre-processed files were then uploaded to FlowJo for gating. EQ beads, Rh (DNA intercalator) and event length parameter were used to determine intact DNA+ve singlets from debris and cell aggregates. Pt live/dead stain was then used to identify live intact singlets. B cell populations were determined by gating on CD45^+^ CD19^+^ populations and then refined further for doublet exclusion using the markers: CD45, CD19, CD8a, CD4, CD3 and CD16a, to determine a ‘True’ CD19^+^ population (**Supplementary Figure 1 (a)**). Further downstream analysis of B cell phenotype was performed in R using modified script from the CATALYST, diffCYT, FLowSOM, edgeR and flowCORE packages which can be found using the bioconductor terminal [https://www.bioconductor.org/packages/release/bioc/vignettes/CATALYST/inst/doc /differential.html](https://www.bioconductor.org/packages/release/bioc/vignettes/CATALYST/inst/doc%20/differential.html).

**Supplementary Figure 1. CyTOF gating of B cells and cluster analysis.**

**(a)** CyTOF gating strategy for the identification of CD19^+^ B cells from peripheral blood mononuclear cells. **(b)** Heatmap representing the median scaled expression of the 19 markers used for B cell clustering.

**Supplementary Figure 2. Proportion of TGF-β^+^ B cells declines following ex vivo stimulation of healthy volunteer and melanoma patient peripheral blood B cells.** Quantification of %TGF-β-expressing B cells from healthy volunteer and melanoma patient peripheral blood either without ex vivo stimulation or following 72-hour culture with 0.1µg/ml CpG or CD40L + 10µg/ml CpG.

**Supplementary Figure 3. Flow cytometric gating strategies used for B cell phenotyping.** **(a)** Gating strategy used for the identification of CD19^+^ B cells from peripheral blood mononuclear cells following ex vivo culture. **(b-c)** Isotype controls were used to set the gates for TGF-β^+^ (b) and PD-L1^+^ (c) B cells.

**Supplementary Figure 4. Distribution of B cell phenotypes following ex vivo stimulation, and dimensionality reduction using the tSNE and FlowSOM Algorithms for lineage analysis of B cells expressing each of IL-10, TGF-β and TNF-α.**

Melanoma patient peripheral blood mononuclear cell suspensions were incubated for 72 hours, with a cytokine stimulation cocktail added for the final 6 hours. **(a)** Quantification of naïve, IgM memory and class-switched (C/S) memory B cells (% of total B cells, phenotypes defined by dimensionality reduction based upon the expression of CD27, IgD, IgM, CD24 and CD38) either without ex vivo stimulation, or following 72-hour culture with 0.1µg/ml CpG or CD40L + 10µg/ml CpG. **(b-d)** Live single CD19^+^ B cells were gated and used for dimensionality reduction. **(b)** TGF-β was detected in B cells in the absence of ex vivo activation. B cells were activated with CD40L + 10µg/ml CpG (ODN 2006) for the detection of **(c)** IL-10^+^ Bregs and 0.1µg/ml CpG for the detection of **(d)** TNF-α^+^ inflammatory B cells. [Left] The FlowSOM algorithm was used to generate six meta-clusters per sample based upon the expression of CD27, IgD, IgM, CD24 and CD38. Heatmaps illustrate marker expression within each cluster (blue = low; red = high). Each cluster is annotated according to the B cell lineage phenotype and the largest three lineage populations are highlighted in bold. [Right] tSNE projections mapping B cells according to the five marker-panel. FlowSOM clusters are indicated by color-coding. Plus (+) symbols indicate degree of expression, where “+” = low or intermediate and “++” = high expression.

**Supplementary Figure 5. Expression of regulatory (TGF-β and IL-10) cytokines and decreased pro-inflammatory (TNF-α) cytokines in the melanoma TME.**

Comparison of TGF-β, IL-10 and TNF-α gene expression in N=461 melanoma lesions and N=558 normal skin tissues.

**Supplementary Figure 6. Immunofluorescence reveals close proximity of CD20^+^ TGF-β^+^ Bregs and CD3^+^ T cells within the melanoma tumor microenvironment.**

Example immunofluorescence images highlight close proximity of CD20^+^ TGF-β^+^ Bregs (yellow) with CD3^+^ T cells (purple) within a melanoma tumor section. DAPI; blue, CD20; green, TGF-β/IL-10/TNF-α; red, CD3; purple. Scale bar = 50µm (main image) and 10µm (inset images).

**Supplementary Figure 7. TNF-α-expressing inflammatory B cells are diminished in melanoma tumors compared to the circulation.**

1. Flow cytometry gating strategy for the identification of CD19^+^ B cells from melanoma lesion single cell suspensions. **(b)** [Left] Flow cytometry plots show gating of CD19^+^ IL-10^+^ B cells from a representative tumor single cell suspension. [Right] No significant difference in the % of total IL-10^+^ Bregs is observed between melanoma patient peripheral blood (N=18) and tumor tissue (N=14). **(c)** [Left] Flow cytometry plots show gating of CD19^+^ TNF-α^+^ B cells from a representative tumor single cell suspension. [Right] The proportion of TNF-α^+^ inflammatory B cells out of total B cells is significantly lower in tumor lesions (N=5) compared to melanoma patient peripheral blood (N=18).

**Supplementary Figure 8. FOXP3 expression among tumor-infiltrating lymphocytes is preferentially localized to T cells, and tumor-infiltrating Tregs express TIGIT.**

**(a)** UMAP visualizations defined by global GEx of single cells pooled from N=12 patient tumors (19944 cells), highlighting [left] distribution of major immune cell subsets (B, myeloid, NK, plasma, and T cells) and [right] FOXP3 expression. **(b)** Tumor-infiltrating FOXP3-expressing cells show expression of PTPRC (CD45), CD247, CD4, CD27, and CCR5, indicative of T-helper cell phenotype. **(c)** Violin plots depicting FOXP3 expression among major immune cell subsets, pooled from N=12 melanoma lesions (19944 cells). Expression is preferentially localized to the T cell population. **(d)** UMAP visualizations defined by global GEx of single cells pooled from N=12 patient tumors (19944 cells), highlighting [left] FOXP3, [middle] TIGIT and [right] combined FOXP3 and TIGIT expression. Color threshold key indicates normalized gene expression for TIGIT (green), FOXP3 (red) and combined FOXP3 and TIGIT expression (yellow). **(e)** Quantification of FOXP3^+^ and TIGIT^+^ cells within a Treg-annotated population, pooled from N=12 melanoma lesions.
