# Supplementary material for: Enriched circulating and tumor-resident TGF-β+ regulatory B cells in patients with melanoma promote FOXP3+ Tregs
Source: Oncoimmunology. 2022 Jul 28;11(1):2104426. doi: 10.1080/2162402X.2022.2104426 (PMC9336482; doi:10.1080/2162402X.2022.2104426)
Supplement: Supplemental Material [file KONI_A_2104426_SM9602.zip › Supplementary_Table_1.docx]

Healthy volunteers

| Variable | CyTOF | ICA |
| --- | --- | --- |
| **Age**, years (mean +/- SD) | 60.7 (17.3) | 50.4 (15.5) |
| Range | 28-88 | 24-74 |
| **Sex (%)** |  |  |
| Male | 3 (23.1) | 10 (52.6) |
| Female | 10 (76.9) | 9 (47.4) |
| ICA = Intracellular cytokine assay |  |  |
