# Supplementary material for: Enriched circulating and tumor-resident TGF-β+ regulatory B cells in patients with melanoma promote FOXP3+ Tregs
Source: Oncoimmunology. 2022 Jul 28;11(1):2104426. doi: 10.1080/2162402X.2022.2104426 (PMC9336482; doi:10.1080/2162402X.2022.2104426)
Supplement: Supplemental Material [file KONI_A_2104426_SM9602.zip › Supplementary_Table_2.docx]

Supplementary Table 2. Melanoma patient cohort clinical characteristics. A

Melanoma patients

| Variable CyTOF | ICA (blood) | ICA (tumour) | IHC | CSA | CPA | TIA | ST |
| --- | --- | --- | --- | --- | --- | --- | --- |
| Age, years (mean +/- SD) 74.4 (8.5) | 55.6 (17.1) | 70.2 (16.0) | 60.7 (18.6) | 53.2 (20.4) | 61.2 (18.5) | 56.3 (11.6) | 63.7 (9.7) |
| Range 57-88 | 25-83 | 35-93 | 31-84 | 25-76 | 38-94 | 33-75 | 53-72 |
| Sex (%)  Male 16 (53.3) | 10 (52.6) | 10 (62.5) | 4 (66.7) | 2 (40.0) | 4 (40.0) | 5 (41.7) | 1 (33.3) |
| Female 14 (46.7) | 9 (47.4) | 6 (37.5) | 2 (33.3) | 3 (60.0) | 6 (60.0) | 7 (58.3) | 2 (66.7) |
| Stage (%)  0(0.0) | 0(0.0) | 1(6.3) | 0(0.0) | 0(0.0) | 0(0.0) | 0(0.0) | 0(0.0) |
| 2(6.7) | 4(21.0) | 3(18.7) | 1 (16.7) | 0(0.0) | 1 (10.0) | 1 (8.3) | 2(66.7) |
| 16(53.3) | 14 (73.7) | 11 (68.7) | 1 (16.7) | 5 (100.0) | 9(90.0) | 10 (83.4) | 1 (33.3) |
| IV 12(40.0) | 1(5.3) | 1(6.3) | 4(66.7) | 0(0.0) | 0(0.0) | 1(8.3) | 0(0.0) |

IC:A = Intracellular cytokine a ssay, IHC: = IirnJunohistochemistry. CSA = Cytokine suporession assay CPA = Cellrilar proliferation assay, TIA = Treg induciion a ssay, S+ = Spatial Irans criotomics

| B |  | | |
| --- | --- | --- | --- |
| Patient ID | Single cell cohort  Tumor location | Disease Stage | Treatment |
| p11 | Subcutaenous | III | None |
| p 12-1 | Subcutaenous | II I | None |
| p13 | Subcutaenous | III | None |
| p15 | Subcutaenous | III | None |
| p16 | Subcutaenous | III | None |
| p 17-1 | Subcutaenous | III | None |
| pd 8 | Subcutaenous | III | None |
| p4 9 | Subcutaenous | III | None |
| p21 | Subcutaenous | III | None |
| p25 | Primary | III | None |
| p26 |  | II | None |
| p3 | Subcutaenous | IV | None |
