# Supplementary figures and images for: Enriched circulating and tumor-resident TGF-β+ regulatory B cells in patients with melanoma promote FOXP3+ Tregs
Source: Oncoimmunology. 2022 Jul 28;11(1):2104426. doi: 10.1080/2162402X.2022.2104426 (PMC9336482; doi:10.1080/2162402X.2022.2104426)

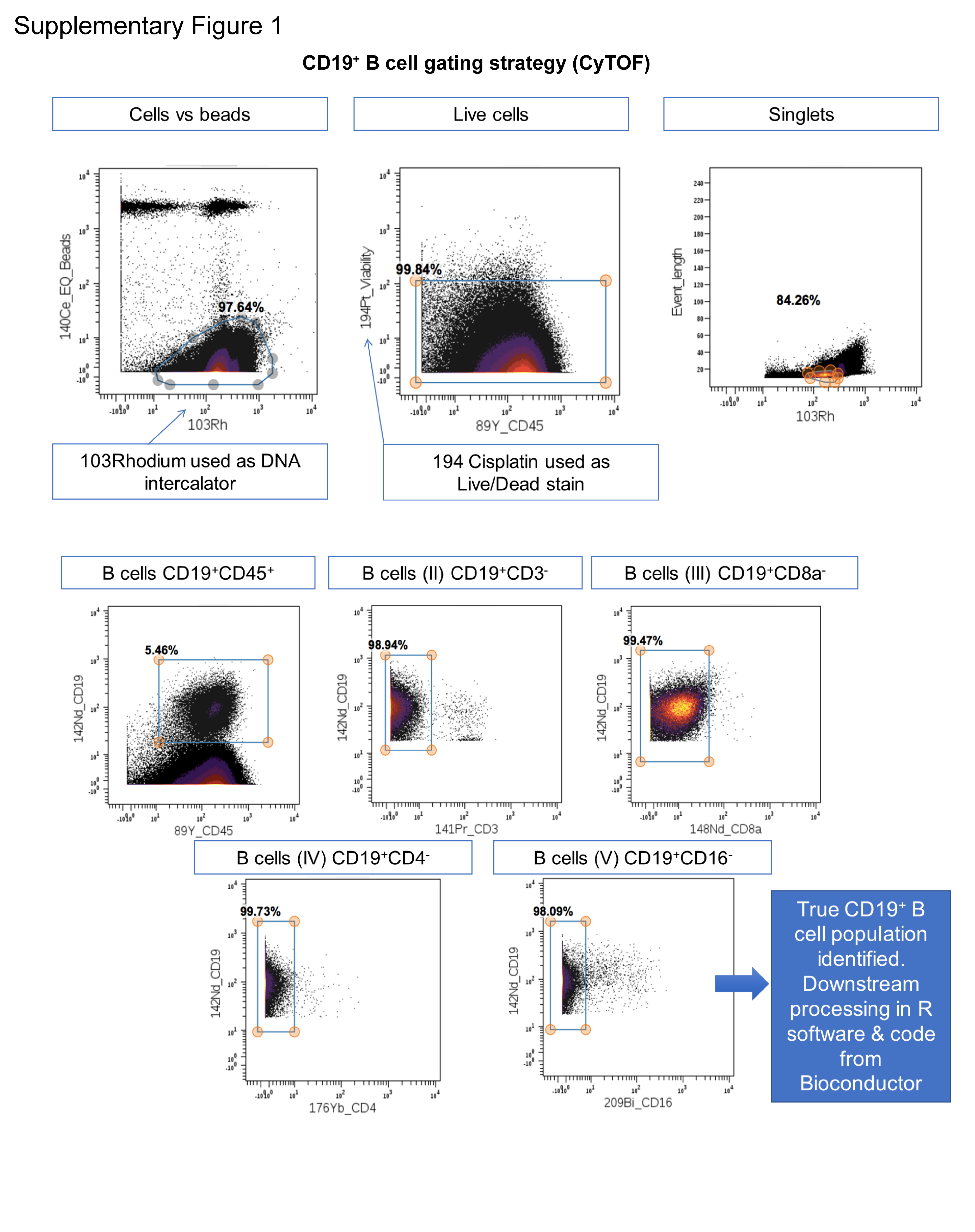

Supplement: Supplemental Material [file KONI_A_2104426_SM9602.zip › Supp_Fig_1.tif]

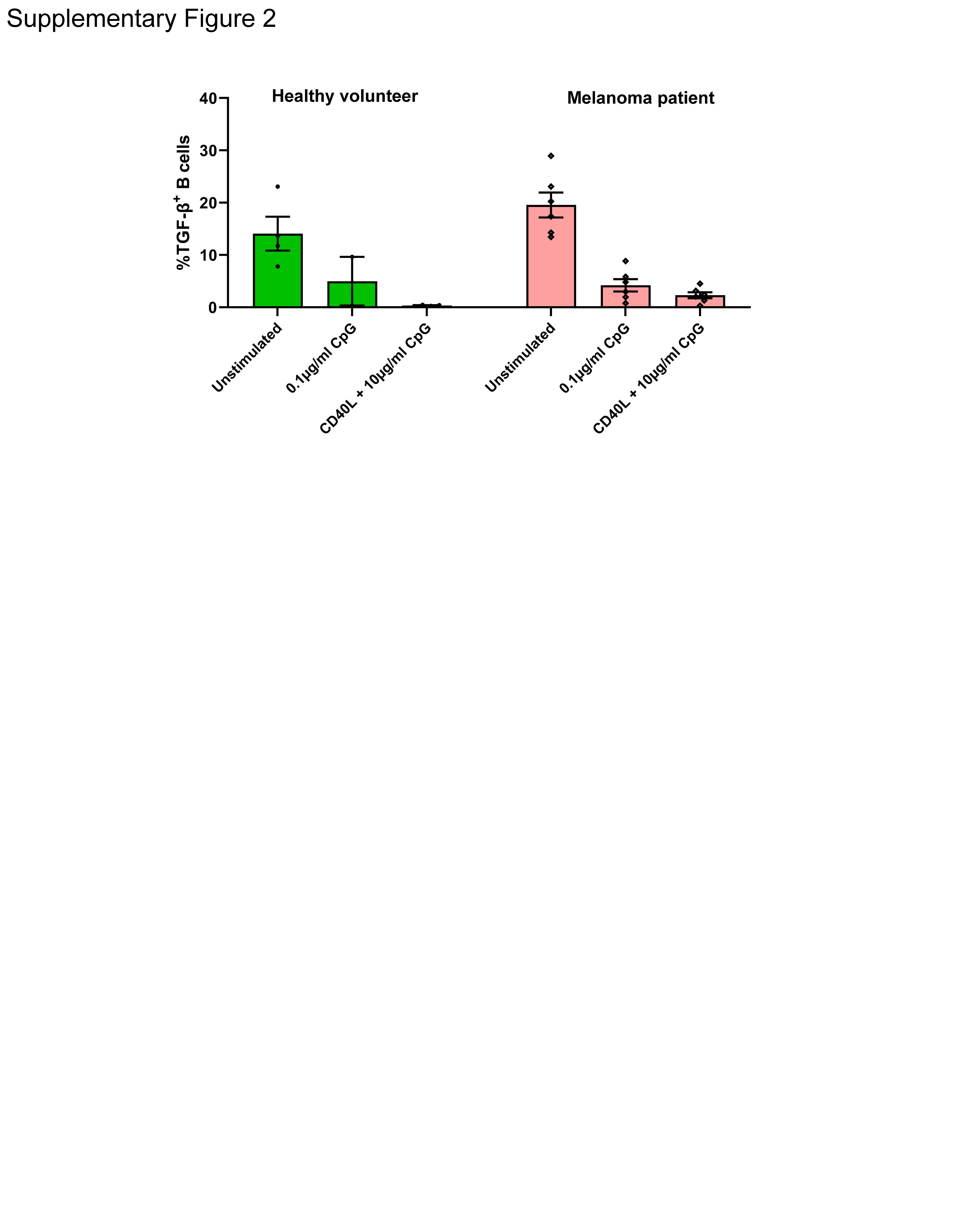

Supplement: Supplemental Material [file KONI_A_2104426_SM9602.zip › Supp_Fig_2.tif]

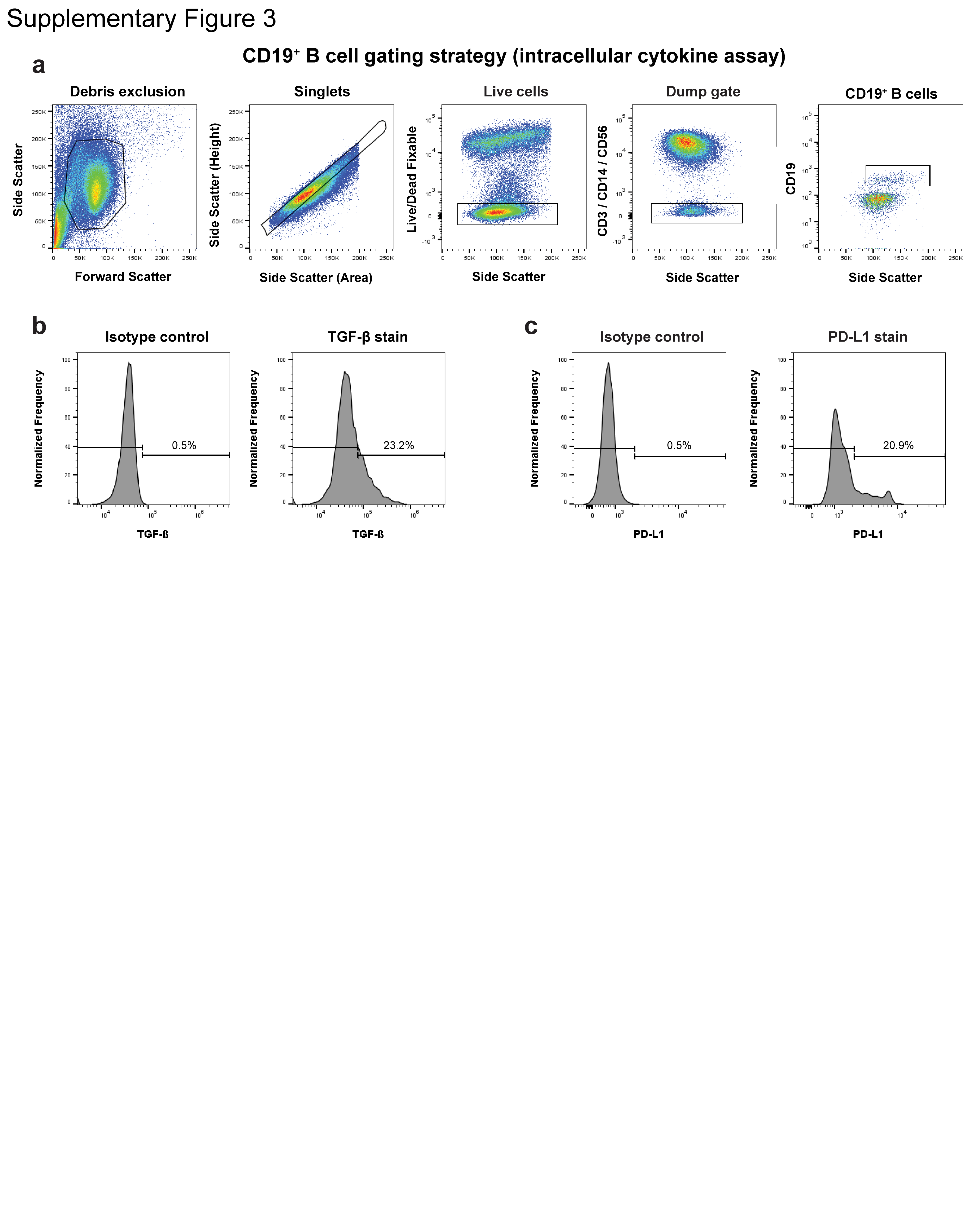

Supplement: Supplemental Material [file KONI_A_2104426_SM9602.zip › Supp_Fig_3.tif]

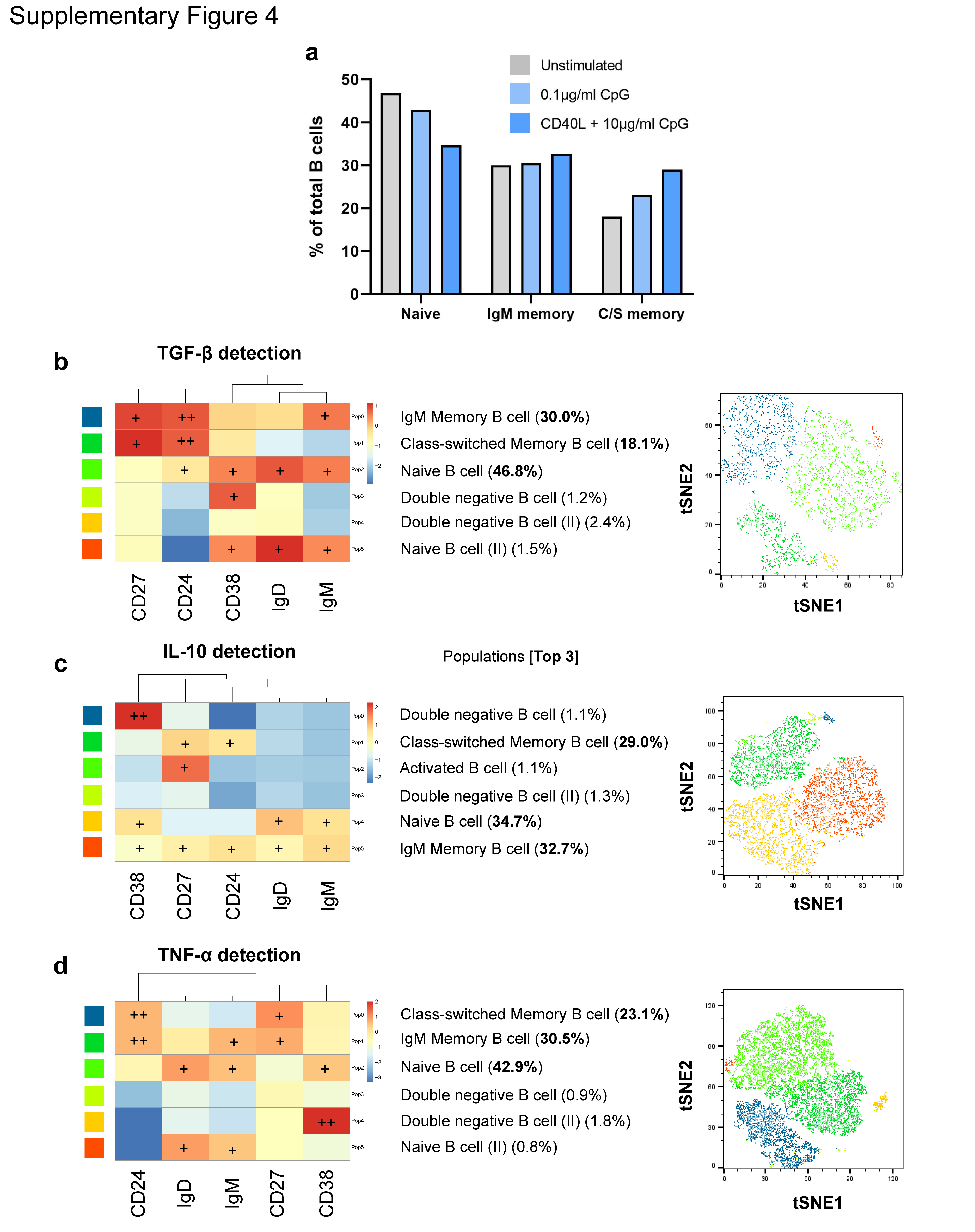

Supplement: Supplemental Material [file KONI_A_2104426_SM9602.zip › Supp_Fig_4.tif]

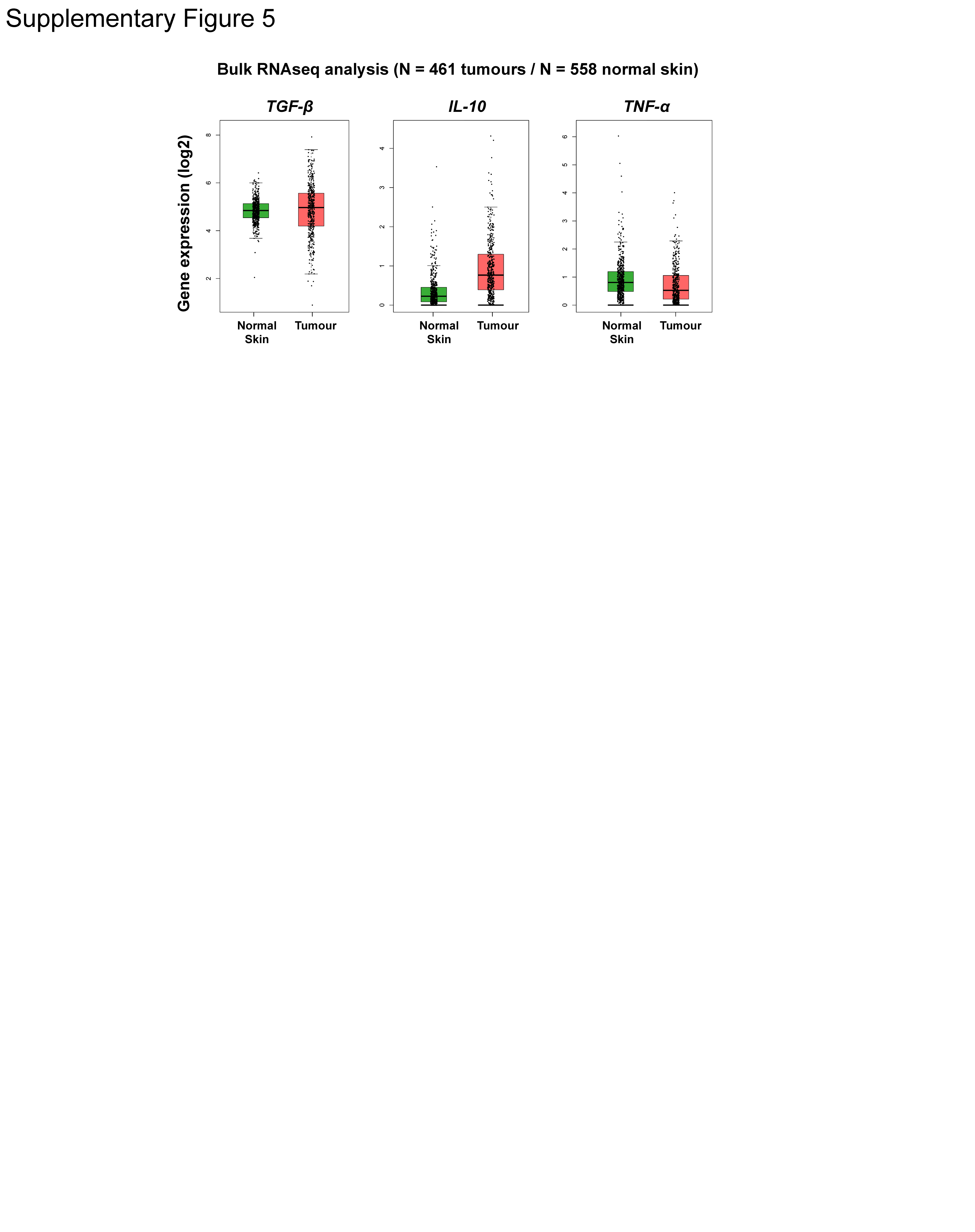

Supplement: Supplemental Material [file KONI_A_2104426_SM9602.zip › Supp_Fig_5.tif]

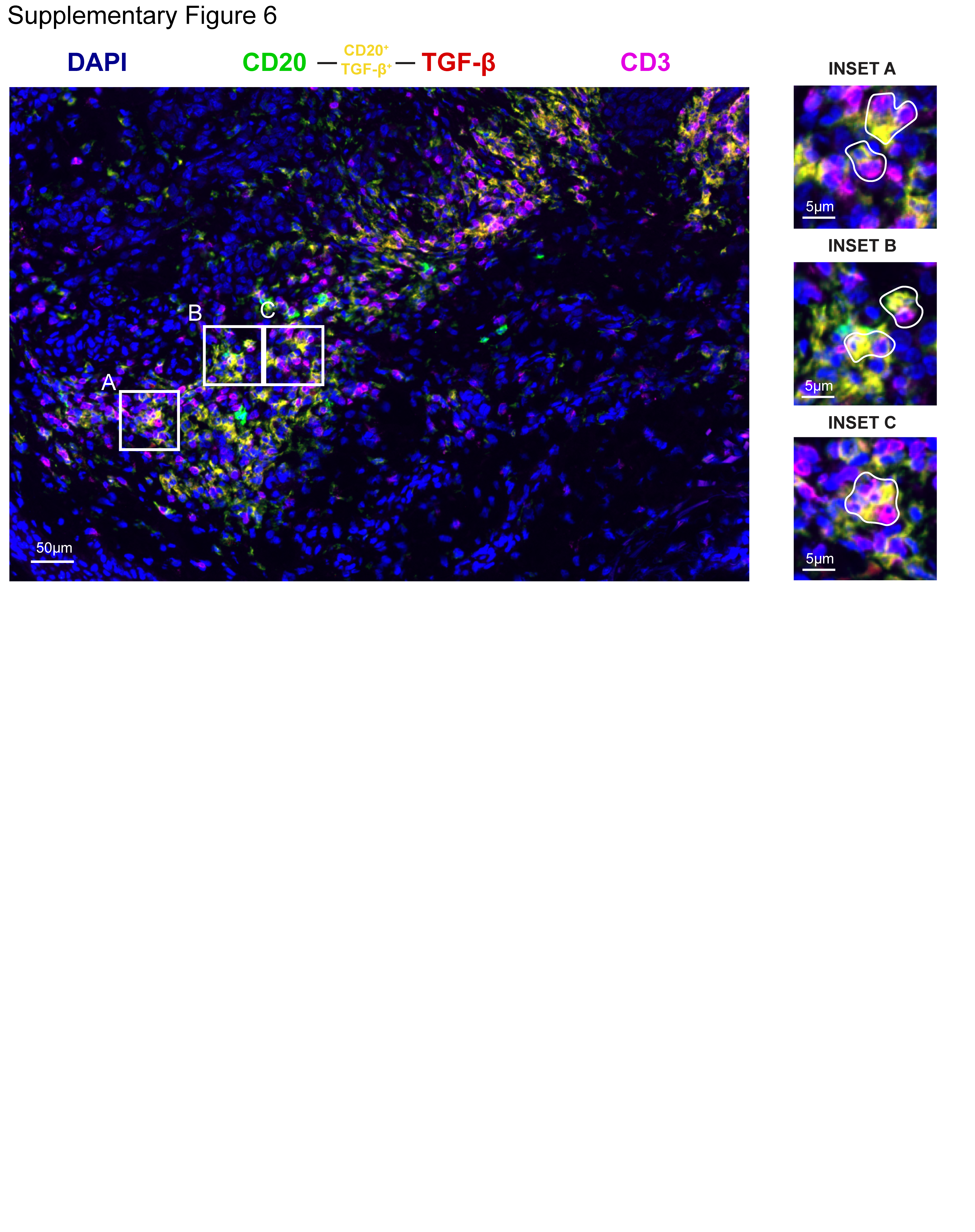

Supplement: Supplemental Material [file KONI_A_2104426_SM9602.zip › Supp_Fig_6.tif]

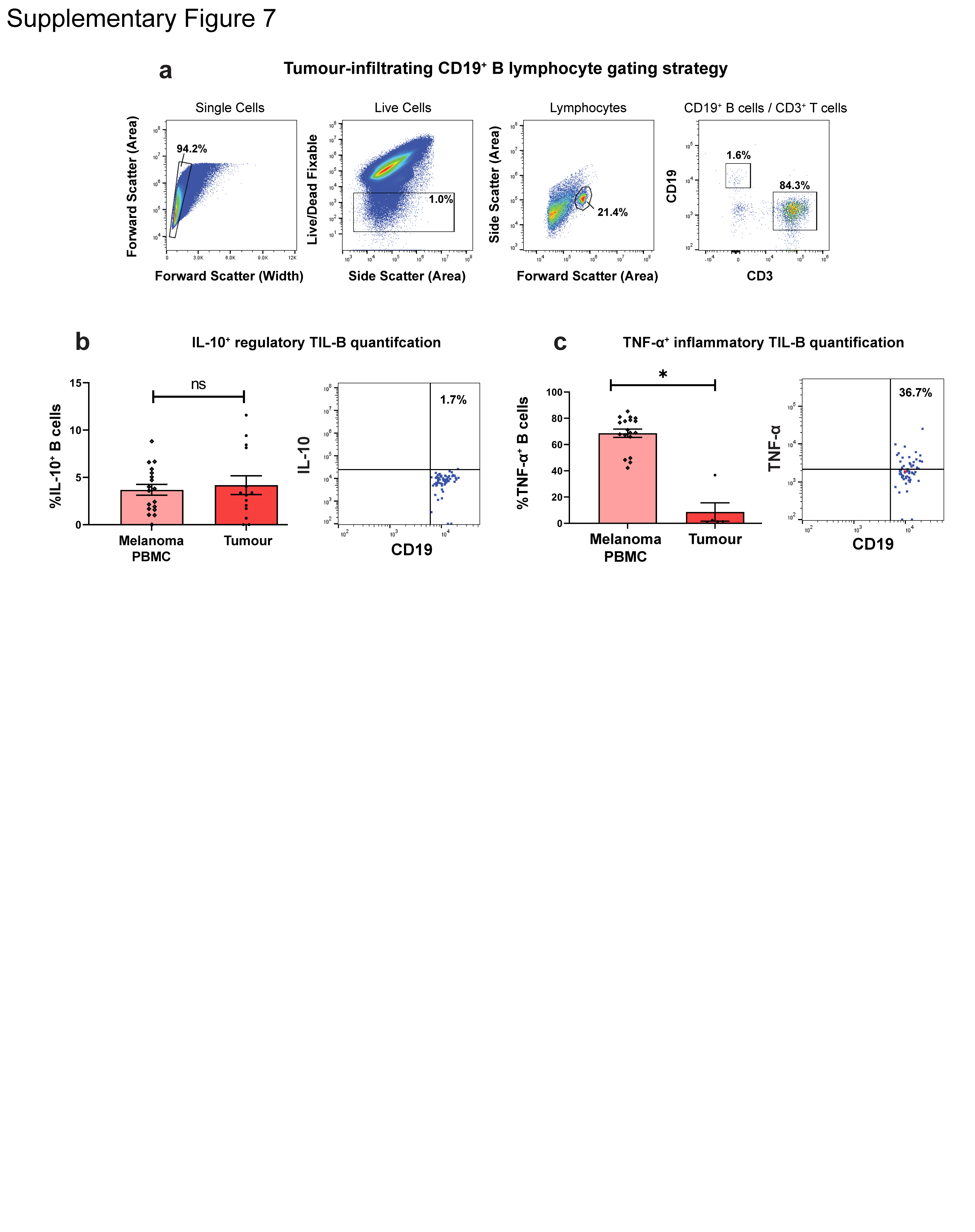

Supplement: Supplemental Material [file KONI_A_2104426_SM9602.zip › Supp_Fig_7.tif]

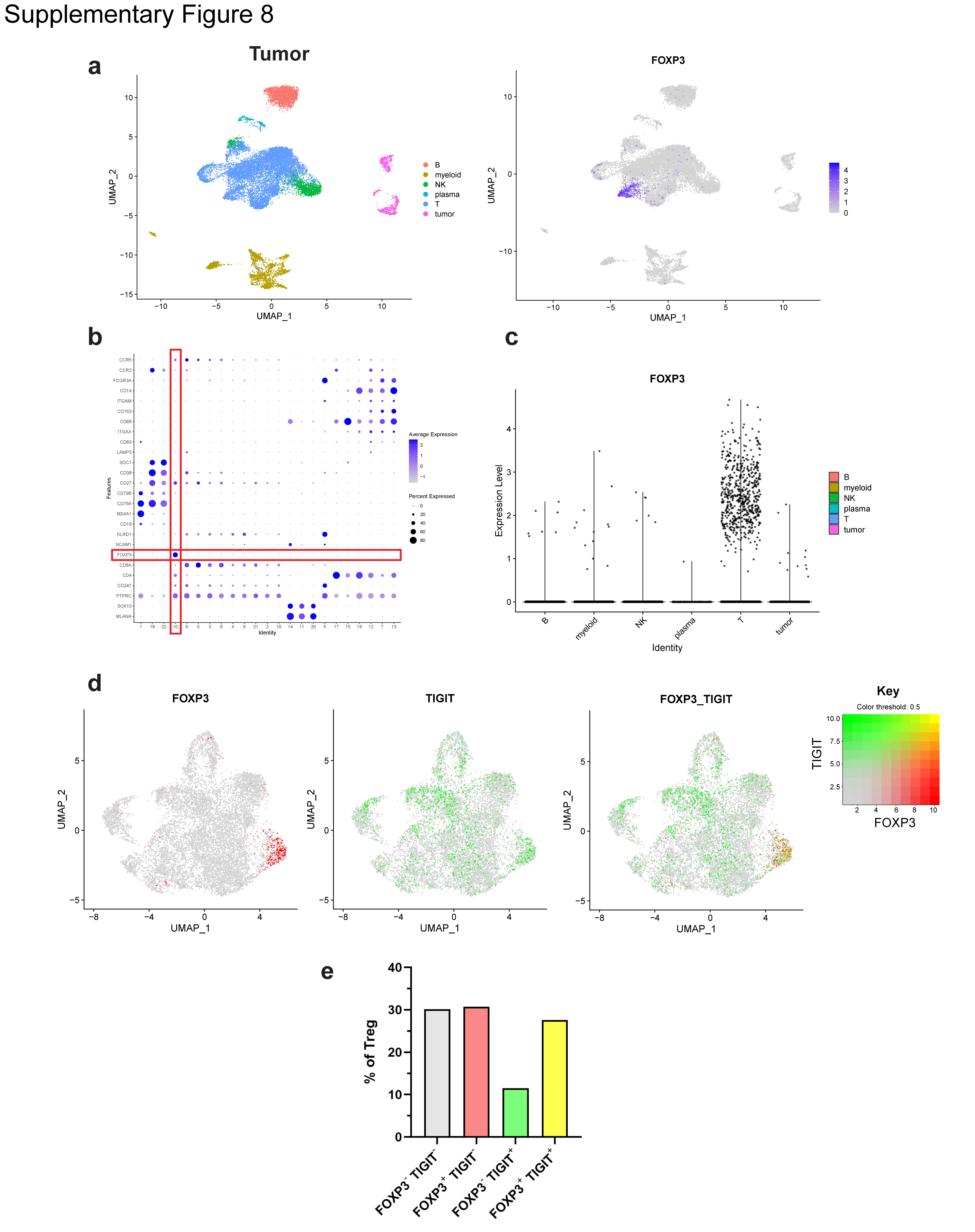

Supplement: Supplemental Material [file KONI_A_2104426_SM9602.zip › Supp_Fig_8.tif]
